# New Products

**DOI:** 10.1155/S1463924684000237

**Published:** 1984


Journal of Automatic Chemistry, Volume 6, Number 2 (April–June 1984), pages 98–118

### New Products fluorescence units in a Microtiter plate matrix print-out.

*Leaflet from Dynatech Laboratories Ltd, Daux Road, Billingshurst, Sussex RH14 9SJ, UK. Tel.: 040381 3381.*

Circle No. 59 on Reader Enquiry Card

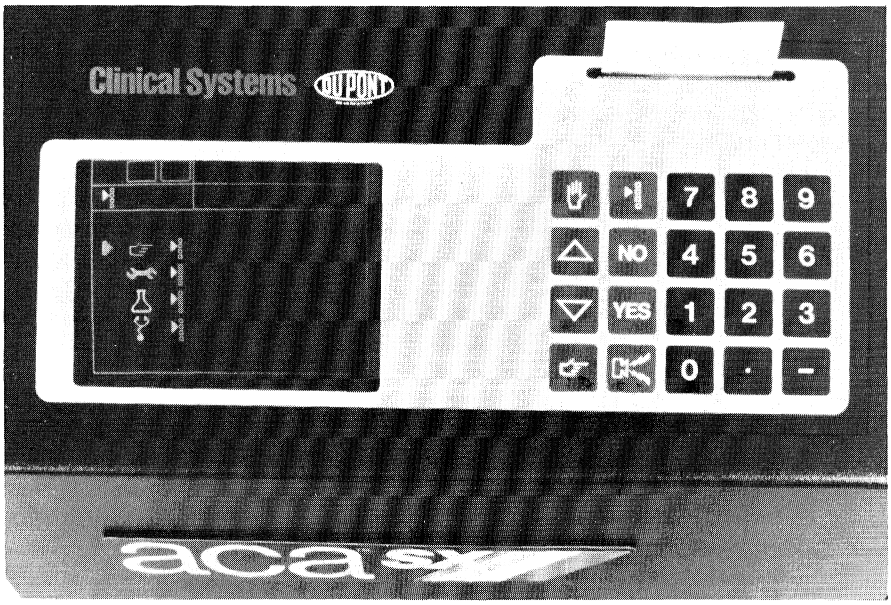

*Du Pont's ACA SX discrete clinical analyser communicates with operators in memorable, easy-to-understand symbols that facilitate training and eliminate language barriers. The instrument's simple keyboard contains only 12-digit entry and eight special function keys.*

### Benchtop clinical analyser

ACA SX (113 cm wide × 69 cm deep × 49 cm high) is a modular, discrete clinical analyser capable of performing 48 tests. The analyser features 'pictorial' (see photograph) communication to simplify training and operation; self-aligning components and self-contained diagnostics make servicing easier.

It is based on its manufacturer's (Du Pont) test pack technology: the test pack contains the reagents needed for each chemical analysis. The ACA SX injects the patient sample into the individual test pack, performs the analysis and prints the results. Results are available in $7\frac{1}{2}$ min. Tests include general and special chemistries, enzymes, therapeutic drugs, coagulation tests and immunoglobulins. Chemistries range from admission tests, such as glucose and BUN, to many specialized diagnostic tests—amylase and ammonia for example. Each test process is completely automatic, controlled by software driving a central computer with a 16-bit microprocessor and seven satellite microprocessors regulating and monitoring every step.

More than 20 additional tests are presently being developed, all of which can easily be added to the ACA SX with a disk change.

*Full information from Du Pont de Nemours International S.A., PO Box, CH 1211 Geneva 24, Switzerland. Tel.: 022 378111.*

Circle No. 58 on Reader Enquiry Card

### Automatic microplate fluorescence reader

The Microfluor reader enables 96 separate assays to be performed simultaneously in 30 s (traditional methods typically take $1\frac{1}{2}$ h). The system is as sensitive and precise as radioimmunoassay, but has none of the problems associated with RIA.

Microfluor measures the relative fluorescence of light emitted by a sample in the Microtiter plate well. Specially formulated low fluroescence Microfluor plates, used in performing fluorescent immunoassays, eliminate background 'noise'. The data is recorded on a separate thermal printer and presented as relative

### Laboratory automation

PALM is short for 'Philips Automated Laboratory Management' system; it is modular and can be tailored to meet precise application needs whilst retaining enough flexibility for subsequent expansion or modification. A DEC PDP 11 forms the main processor, receiving input from a range of analytical instruments and input/output terminals. Standard software allows the computer to be linked into a larger data-processing complex or to be connected on-line to a mainframe unit for centralized storage or automated plant control.

Of course many of Philips's computer-controlled instruments form ideal intelligent inputs to PALM—they can be linked directly to the central PDP 11 or connected via an intermediate mini- or microcomputer dedicated to one or more instruments. The system can also incorporate equipment from other suppliers, and provide for manual data entry.

PALM was developed principally for industrial process control and quality assurance and provides the fast information feedback essential for plant efficiency and high throughput—the same criteria apply to many research and development laboratories. The software range includes all the modules necessary for day-to-day sample analysis, together with storage and retrieval of comprehensive management information on laboratory and plant performance.

It is easy to use, with simple menu presentations giving access to a range of functions. Extensive error messages effectively safeguard against mistakes and 'help' messages can be called up.

Managers can quickly set up and modify laboratory routines to suit their individual working practices, but security procedures prevent unauthorized access to sensitive data.

Urgent results can rapidly be made available by dividing the data and storing it in separate active and passive data-bases; the active data-base contains full details of up to 4000 current samples. The system automatically progresses these data packages through the laboratory from the moment their identities are typed in at any of the system's work terminals. Samples awaiting operator attention are signalled by the computer. Backlog reports can also be generated, showing the accumulated work-load per instrument or for the laboratory as a whole. Individual analysis results automatically integrate to collate complete sample reports, which may include special modulus calculations or limit checks verifying that specifications are being met. A sample can be removed from the active data-base only when the user-defined actions have been completed; it is then transferred to the passive data-base for incorporation into management reports, archiving or deletion.

A variety of reports can be derived from the stored information, enabling managers to observe trends in materials and processors, monitor laboratory efficiency and maintain close control of costs.

The PALM scheme includes a number of standard form-generation routines which can be supplemented by packages available from DEC. The programs are written in FORTRAN 77 and experienced users can readily create their own software.

*More information from Pye Unicam Ltd, York Street, Cambridge CB1 2PX, UK. Tel.: 0223 358866.*

**Circle No. 60 on Reader Enquiry Card**

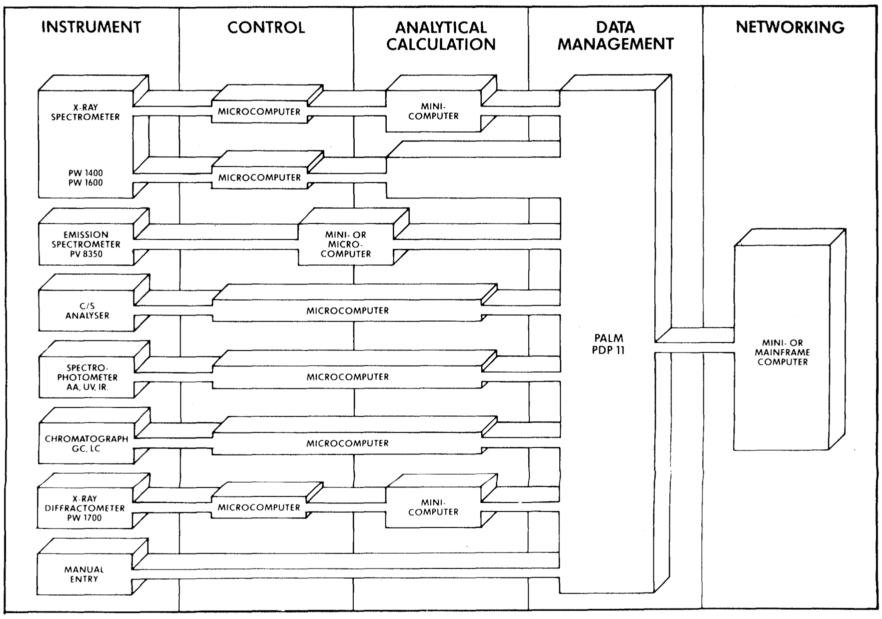

*Philips Automated Laboratory Management system (PALM). 'A totally integrated software concept covering all aspects of the analytical operation—from sample identification to information management, reporting and archiving' (Pye Unicam Ltd, UK).*

### Electronic analytical balance

The AE 100 has been newly added by Mettler Instrumente to their line of electronic analytical balances. The AE 100 has a weighing range from 0 to 109 g and a readability of 0·1 mg. The single control bar assures maximum operating convenience and the weighing pan is positioned only millimeters above the counter-top for fast, easy weighing-in. The weighing chamber is roomy and is accessible from both sides and the top. An optional data output makes it possible to reliably transfer measuring values to a printer, calculator or computer.

*Details from Mettler Instrumente AG, CH 8606 Greifensee, Switzerland.*

**Circle No. 61 on Reader Enquiry Card**

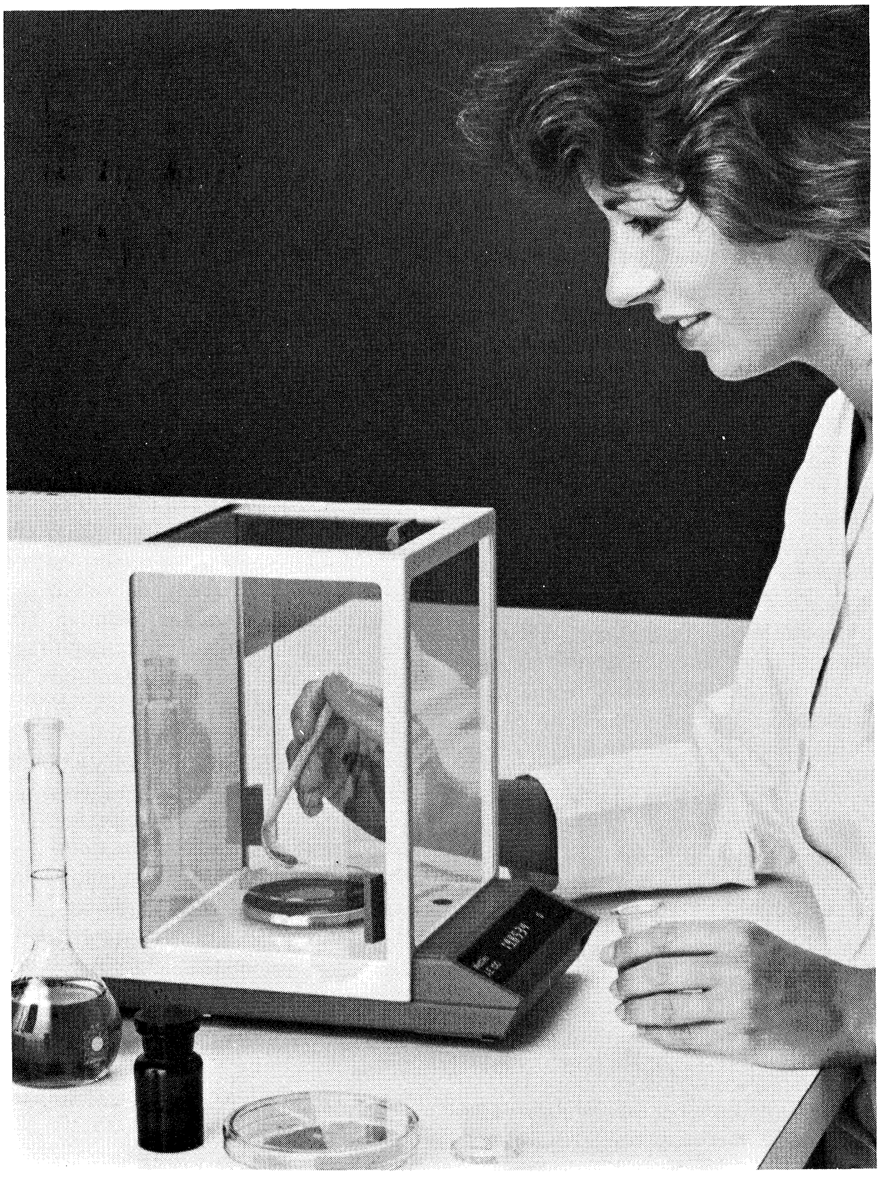

*The AE 100 which Mettler consider will make mechanical balances obsolete. As long as a capacity of 109 g is sufficient, it is an attractive proposition for organizations working within tight budgets—it is recommended for schools.*

### Ultrolab in Switzerland

The Swedish-based manufacturer of clinical chemistry analysers, Ultrolab, has set up a subsidiary in Switzerland. Further expansion is expected in the near future.

*The Swiss operation is at Steinhauserstrasser 21, CH 6330 Cham.*

**Circle No. 62 on Reader Enquiry Card**

### I-R 100 awards

Two of Instrumentation Laboratory's 1983 products have won I-R 100 awards: the Statsep rapid plasma separator and the Video 22 atomic absorption spectrophotometer. I-R 100 awards are made by the *Industrial Research and Development* journal for the 100 most significant technological advances in the year.

The Statsep uses membrane filtration to separate plasma from whole blood in 1 min, in contrast to the process of centrifugation which requires approximately 10 min. The quick production of plasma made possible by the Statsep allows the determination of anticoagulant levels in the blood during open-heart surgery in as little as 5 min; it is also a valuable tool in emergency and operating rooms during severe bleeding, as well as for general laboratory use for 'stat' testing.

The Video 22 atomic absorption spectrophotometer is a dual-channel, double-beam atomic absorption system capable of simultaneously determining two elements in a single sample. The Video 22 is equipped with the Smith-Hieftje background correction system which separates electronic noise from the signal being measured. The product, a winner in the I-R 100 'analytical instruments and components' category, was developed by Stanley B. Smith, Jr., IL Analytical Instruments Division, and Gary M. Hieftje, Professor of Chemistry, Indiana University; the Smith-Heiftje innovation eliminates many of the problems associated with other background correction systems, and at a lower cost.

*Information about the Statsep and Video 22 from Instrumentation Laboratory, Inc., 113 Hartwell Avenue, Lexington, Massachusetts 02173, USA. Tel.: 617 861 0710.*

**Circle No. 63 on Reader Enquiry Card**

### Detector cells

A range of electrochemical cells from ESA (who make the Coulochem detector) is to be sold in UK by Severn Analytical.

New analytical cells for the system include the 5011 high-sensitivity model with one coulometric electrode and one high-efficiency amperometric electrode. Detection of catecholamines as low as 500 femtograms, with a signal to noise ratio of 8-to-1, has been reported with this cell.

The 5012 screened wall-jet cell again uses a single coulometric porous graphite electrode but follows this with a wall-jet design electrode composed of a combination of gold, silver, nickel and graphite. These materials enhance selectivity of the cell by allowing detection of compounds not active on graphite alone.

For pretreatment, a conditioning cell is available, placed between the column and the analysis cell. This provides an additional screen or redox step for complex samples.

*Details from Severn Analytical, 36 Brunswick Road, Gloucester GL1 IJJ, UK.*

**Circle No. 64 on Reader Enquiry Card**

### Water purification

A reverse osmosis unit has been added by Fisons to the Fistream range of water-purification products. (Reverse osmosis is a cheap way of pretreating tapwater for further purification by more complete methods such as distillation and deionization.)

The RO60 will deliver 60 l/h of good-quality purified water. A purity meter displays the water quality in microSiemen/cm; it also displays the salt rejection rate which indicates the performance of the membrane.

An audible/visual alarm prevents unacceptable quality water being drawn; the unit is also fitted with a 2 min delay after switch-on to enable high-quality water to be attained before being delivered.

Periodic membrane cleaning is a feature—automatic controls ensure correct dilution of the cleaning solution and subsequent rinsing. And noise is virtually eliminated by a heat exchange jacket around the high-pressure pump; this also preheats water to the membrane to improve the output performance, particularly during cold weather.

The RO6O can be coupled directly to other purification equipment or it can deliver to a holding tank. In the latter condition, a float switch shuts down the unit whenever the reservoir is full.

*Details from Fisons PLC, Scientific Equipment Division, Bishop Meadow Road, Loughborough, Leicestershire LE11 0RG, UK. Tel.: 0509 231166.*

**Circle No. 65 on Reader Enquiry Card**

### New generation TLC scanner

CAMAG stress three aspects of their TLC Scanner II.

The machine is versatile, having been designed for scanning in both the absorption and fluorescence modes. All of the light sources required (mercury, deuterium and tungsten lamps) are built in along with their power supplies.

The TLC Scanner II is easy to operate: scanning parameters are entered by dialogue with the microprocessor which controls the sequence of operations. Nine memories are available to store scanning programs, which can then be called up by a single command. All controls are neatly arranged on the front panel. Built-in UV lighting of the pilot image of the scanning slit make accurate positioning of the sample easy. Scanning sensitivity and zero setting are adjusted automatically.

Signal-processing capabilities are excellent: a wide range of peripherals is available for further processing of raw data. A CAMAG version of the SP 4270 integrator is offered as standard—it features such TLC-oriented integration programs as multi-level calibration and automatic calculation of standard deviations. A desk-top computer (HP 9816) can be used for computer-controlled evaluation of chromatograms.

*More information from Ch. Gfeller, CAMAG, Sonnenmattstrasse 11, CH 4132 Muttenz, Switzerland. Tel.: 061 613434.*

**Circle No. 66 on Reader Enquiry Card**

### New book

*Luminescent Assays: Perspectives in Endocrinology and Clinical Chemistry* (Techmation, 1984, edited by M. Serio and M. Pazzagli) is the proceedings volume of the Serono Symposia on Luminescent Assays which were held in Florence in July 1981. The book has 31 chapters and provides a good overview of current work in luminescence, covering assay methodology for numerous compounds which include HLA antigens, cytotoxic antibodies, genotoxic agents, haemoglobin, steroids and hormones. Immunoassays and competitive binding assays are discussed. Applications in studies of energy metabolism, lipolysis, catecholamine activity, sperm viability and phagocytosis are also included.

*The publication is priced at £28·50 from Techmation Ltd, 58 Edgware Way, Edgware, Middlesex HA8 8JP, UK. Tel.: 01 958 3111.*

**Circle No. 67 on Reader Enquiry Card**

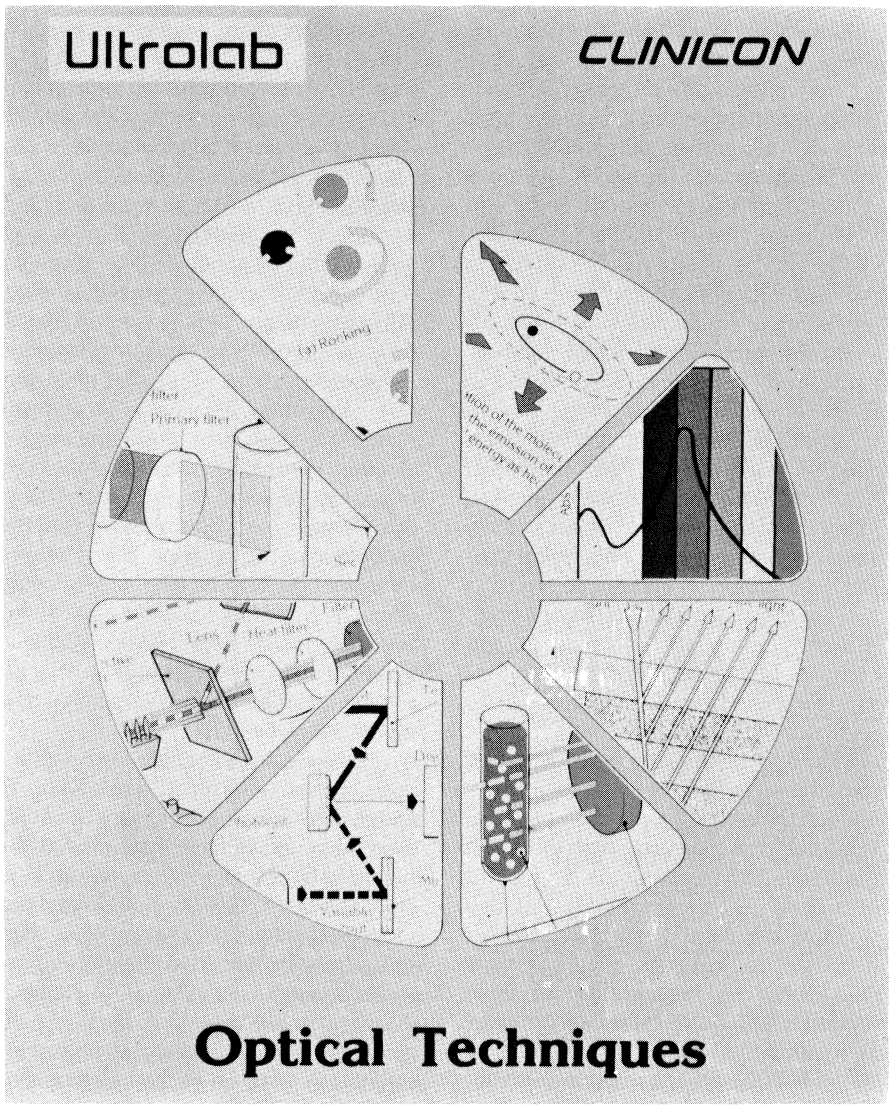

*A multi-author book which Ultrolab/Clinicon commissioned because they needed a training manual to explain the subject in simplified terms to staff and customers. Schools, universities and hospitals have expressed an interest in 'Optical Techniques'. It costs SKr 50·00 or £5·00. Orders to: Ultrolab AB, Box 20032, S-161 20, Bromma, Sweden; or Ultrolab Ltd, PO Box 75, Broadway, Bebington, Wirral L63 5RQ, UK.*

**Circle No. 68 on Reader Enquiry Card**

### Spectrophotometer

A double-beam UV/Vis spectrophotometer was announced earlier in the year by Jasco International. All control functions are performed by microprocessor and spectra can be displayed on the CRT or printed-out on an integral printer/plotter. Menu-driven software provides 12 methods of operation from the 'Spectrum' mode, through 'Quantitative analysis' to 'Data processing', but does allow the operator the freedom of parameter selection at every stage, or the choice of accepting the values pre-set for that particular method.

Peak seeking, derivatives, smoothing, ratios and enzyme analysis are possible, data are stored on floppy disk. Personal programs can be written in BASIC or FORTRAN with the addition of a full keyboard module.

A range of quick-change accessories enables the user to adapt the instrument to each specific application so the benefits of the micro are not restricted by mechanical limitations.

*Literature from the product's distributor: Lea Scientific Ltd, 48 Ramsons Avenue, Conniburrow, Milton Keynes, Buckinghamshire MK14 7BJ, UK. Tel.: 0908 606414.*

**Circle No. 69 on Reader Enquiry Card**

### New HPLC columns

Perkin-Elmer's latest 3 × 3 HPLC columns (so-called because they are 3 cm long and use 3 micron packing materials) are cheaper than most standard columns. However, they are described as offering substantially reduced analysis times and lower solvent consumption, as well as higher efficiency and increased mass sensitivity. The short column length of the 3 × 3s eliminates the high back-pressures and reduced column life sometimes found with other microparticulate columns.

The columns can be used with most LC systems, but to obtain their full capabilities, optimized instrumentation is necessary. The combination of the Perkin-Elmer Series 4 solvent-delivery system, LC-85B detector with a 1·4 $\mu$l flowcell and 3 × 3 columns provides extremely fast analyses—up to 10 times quicker than conventional LC—with no loss in column performance under virtually all chromatographic conditions.

*For further information contact Perkin-Elmer Ltd at Post Office Lane, Beaconsfield, Buckinghamshire HP9 1QA, UK. Tel.: 04946 6161.*

**Circle No. 70 on Reader Enquiry Card**

### Analysis literature

Four leaflets are available from Perkin-Elmer describing accessories for use with the company's Model 240C elemental analyser:

(1) Carbon-in-steel/refractories analysis accessory: for analysing steels, metal alloys and highly refractive substances (for example silicon carbide and tungsten carbide) for carbon content.
(2) Mineral carbonate analysis accessory: for determining organic and inorganic carbon in substances such as oil shales, ocean sediments, soils and rocks.
(3) Liquids and unstable materials sample-handling systems: allow volatile substances such as ethyl ether, gasoline and hydrated materials to be handled.
(4) Oxygen analysis accessory: for determining oxygen content in pharmaceuticals, polymers or energy-related materials, for example oils, petroleum and by-products.

*For a free copy of the leaflets contact Perkin-Elmer Ltd, Post Office Lane, Beaconsfield, Buckinghamshire HP9 1QA, UK. Tel.: 04946 6161.*

**Circle No. 71 on Reader Enquiry Card**

### Sample processor

Prep I, an automated sample processor, was designed for operational convenience and simplicity. It includes a control and program selection panel, a parameter display, solvent storage, a centrifuge with a bidirectional rotor, and disposable sample cartridges that eliminate contamination problems. The Prep I centrifuge uses a 12-place rotor which holds a specially designed four-part cartridge consisting of sample reservoir, resin bed, wash-effluent cup, and sample-recovery cup.

A sample is loaded and then rotated for a predetermined period. Liquid passes through a resin bed which adsorbs compounds of interest. A wash cycle then rids the column of unwanted sample. Washings are caught in an effluent cup and can be discarded or saved for other analyses as desired. Next, rotor direction is changed and eluting solvent is dispensed to the column bed to flush samples of interest into a recovery cup. The sample solution may be used directly on any analytical instrument, or it can be dried by warm air evaporation.

Du Pont's Prep I is being promoted as providing significant time and labour savings over conventional liquid–liquid extraction techniques: manual extraction of 12 theophylline samples from serum, for example, may take only 30 min: however, extraction of 12 catecholamines may take up to 2 h. With the Prep I both separations can be done automatically in 16 min, and microprocessor control assures exact reproducibility of cycles with no operator attention. This not only reduces work required for sample preparation, but it is more precise than manual extractions.

Several cartridges are available for analytical method development or routine sample preparation. These include two types of lipophyllic cartridges, anionic and cationic resin cartridges for removing high polar or charged materials from aqueous solution.

The Prep I is compatible with Du Pont 8800 liquid chromatography instruments.

*For further information contact Du Pont de Nemours International S.A., PO Box CH 1211 Geneva 24, Switzerland. Tel.: 022 378866.*

**Circle No. 72 on Reader Enquiry Card**

### Quality assurance

The first Beckman RTQA system (Real Time Quality Assurance Programme) has been installed at the Department of Chemical Pathology, University College Hospital, London, which handles almost 1M requested test results a year. The system centres on Beckman Decision liquid chemistry controls and includes a TRS 80 micro and serial printer. It monitors the results from the Beckman Astra-8 clinical chemistry analyser, a Technicon SMAC system with 18 channels, and a Cobas centrifugal analyser. The program provides for keyboard entry of data, the processing of results and a commentary about the entered results following comparison with a data-base supplied by the laboratory. Each entry is compared with the last result entered for that test and a check is made with the performance of two other levels of the same analyte, followed by an up-to-date running mean, standard deviation and coefficient of variation calculation. Accuracy and precision bias are calculated and linearity checked. A trend estimation for precision and accuracy is calculated and charts of results for periods up to a month can be plotted for any test.

The system can also provide action response in the form of messages describing action taken on a particular control result failure. A long-term analysis of such results can be carried out and, at the end of each month, a summary diskette is used to store the data. Particular dates or periods of control results can be simply re-examined by reference to the daily diskettes. Added convenience is provided in a substantial reduction on previous methods in the space needed in the laboratory to store data collected, and in the speedy location of any past data needed.

The system, which offers significant benefits in the form of confidence in results, speed of reporting and ease of handling and storing data, should be of interest to many clinical chemistry laboratories for the management of information relating to quality control on instrumentation and reagents.

*Further details from Beckman-RIIC Ltd, Progress Road, Sands Industrial Estate, High Wycombe, Buckinghamshire, UK. Tel.: 0494 41181.*

**Circle No. 73 on Reader Enquiry Card**

### Electrochemical analysis

The systems available from Radiometer (V. A. Howe & Co. is the sole UK agent) have been further expanded with the ISS820 ion-scanning system and the ION 85 ion analyser.

The *ISS820* is based on a new analytical method for the determination of trace metals; the method depends upon the reduction of metal ions at a glassy-carbon electrode by an electrolysis process, during which the metal ions in the sample are pre-concentrated by amalgamation with a thin mercury film on the working glassy-carbon electrode. After pre-concentration, the electrolysis is stopped, and the metals are then oxidized and stripped from the amalgam film during the scanning period. The resulting potential-versus-time curve is automatically recorded and has the form of a redox tritration curve. The order in which the metals are stripped from the amalgam film is a function of the redox potential of each metal and provides a qualitative identification of the metals present. The time interval between two consecutive equivalence points is proportioned to the concentration of a given metal in the solution. The ISS820 is a simple, easily operated recording system for the analysis of traces of heavy metals. Concentrations from 10 mg/l to 0·2 $\mu$g/l can be determined. Simultaneous analyses are possible.

The *ION 85* is suitable for precision measurement and routine work with pH and ion-selective electrodes and handles all modern measurement techniques for pH and specific ion determination. It is compatible with all the liquid-handling sections of Radiometer's titration system so it can be integrated into an automatic ion-analysing system. The ION 85 has a 20-character alphanumeric display which guides the operator step-by-step through calibration, and shows the measurement in one of five modes (the instrument measures mV, pH, pX temperature and ion concentration). It offers direct potentiometry where the concentration of an unknown is determined after calibration with standard solutions; or ion concentration may be determined by indirect methods such as standard addition, analate addition and the Gran's plot technique (all calculations associated with these methods are performed automatically). Calibration data are independently stored, thus eliminating the need to recalibrate when changing mode; a permanent memory retains all the calibration data when the instrument is off.

*More information about Radiometer products in the UK from V. A. Howe & Company Ltd, 12–14 St. Ann's Crescent, London SW18 2LS. Tel.: 01 874 0422.*

**Circle No. 74 on Reader Enquiry Card**

### Infra-red moisture monitor

MM44 (Infrared Engineering Ltd) is a balanced reference version of the MM4 instrument. Its greater sensitivity and selectivity over the MM4 have been achieved with an additional reference wavelength. The MM44 is a non-contacting on-line instrument for measuring the moisture content of materials during continuous production processes. It can be employed to measure moisture in natural granular substances (iron oxides and milk powder for instance), as well as in manufactured products in the form of continuous webs (paper and plastic film). Alternatively, the MM44 can be calibrated to indicate the weight, or thickness, of coatings applied to substrates, or other moisture-related characteristics of a wide variety of materials.

Moisture content can be measured over a considerable operational range: 1 to 90%; below 10%, moisture content readings are accurate to within ±0·1% and above 10% accuracies between ±0·2% and ±0·3% are achieved.

It is temperature tolerant, and moisture readings are not affected by changes in ambient lighting levels. Variations in particle size and colour of the product being gauged also have little influence on readings.

A low-voltage cable connects the two units comprising an MM44: the measuring head and the control and display unit. The measuring head can be fitted with an air-purge unit to keep its viewing aperture clear of dust and dirt. The head weighs 8 kg and measures 280 mm × 200 mm × 125 mm. A $3\frac{1}{2}$ digit display is incorporated in the control-unit which weighs 14 kg and measures 450 mm × 300 mm × 175 mm.

The instrument is of modular construction and has been designed with self-servicing by replacement in mind. LED indicators and test switches within the control unit provide diagnostic facilities for locating faults.

The MM44 can be used on its own, or as a sensor in a fully-automatic control system. Power consumption is 75 W. Optional features include high/low alarms, gauge-failure warning and the provision of a calibration memory containing six pre-set values selectable by push-button.

*More information from Infrared Engineering Ltd, Galliford Road, The Causeway, Maldon, Essex CM9 7XD, UK. Tel.: 0621 52244.*

**Circle No. 75 on Reader Enquiry Card**

### H-P software

LABSAM/3350 is a software package recently introduced by Hewlett-Packard to improve laboratory productivity and information management. It was designed to manage and organize the flow of samples through a laboratory and it can be adapted to suit most organizations. The package's primary functions include: sample log-in, result acquisition, validation and reporting. LABSAM is intended for use with the H-P 3357 Laboratory Automation System.

*Further information from the Enquiry Section, Hewlett-Packard Ltd, Eskdale Road, Winnersh, Wokingham, Berkshire RG11 5DZ, UK.*

**Circle No. 76 on Reader Enquiry Card**

### 'Photodetection Information Service' paper

'A Comparison of the Performance of Photomultiplier Tubes and Silicon Photodiodes', is the latest in a series of brochures from Thorn EMI and deals with the advantages in application of the two types of light detectors and makes comments about options where a performance overlap occurs. The paper is intended as an aid to equipment designers and scientists engaged in all fields of light measurements.

Thorn EMI Electron Tubes Ltd is a world leader in the development, manufacture and marketing of high-technology light-sensing and light-imaging devices, and is part of the Measurement Division of Thorn EMI Electronics.

*Free copies from the Sales Office THORN EMI Electron Tubes Ltd, Bury Street, Ruislip, Middlesex HA4 7TA, UK. Tel.: 08956 30771.*

**Circle No. 77 on Reader Enquiry Card**

### Theophylline test pack for AcA

A new clinical test from Du Pont has the potential for becoming the first major commercial use of monoclonal antibodies in health care. The test, used with the 'ACA' automatic clinical analyser, is for monitoring the level of the drug Theophylline in the blood serum of patients being treated for acute or chronic asthma: dosage adjustment is critical in such treatment. The use of monoclonal antibodies is a relatively new technique involving the modification of cells in the laboratory to produce a new, unique cell line. These cells produce specific antibodies which can be used in test packs as the reagent or reacting material. The monoclonal antibodies are produced by NEN, a Du Pont subsidiary located in Boston.

*Details from Du Pont Nemours International S.A., PO Box, CH 1211 Geneva 24, Switzerland. Tel.: 022 378111.*

**Circle No. 78 on Reader Enquiry Card**

### Dispensers and fillers

The Hamilton range of dispensers, which has been very successful in the industrial market, has been expanded recently with the introduction of the Aquarius Digital Pumping System. The system is intended for users needing higher volume liquid handling than that provided by the company's other machines.

It is a complete pumping system with pneumatic foot switch, hand probe and flexible arm system which may be located in any convenient position. The instrument is controlled by digital electronics, giving the operator choice of single-step, auto-dispense and continuous-flow modes. In the single-step mode the volume selected on the volume encoder is dispensed each time a start signal is given; the number of steps dispensed is shown on the LED, which may be reset to zero at any time. The automatic dispense mode provides a preselected program of volume, time interval and flow rate for the required number of steps. The display shows the number of the step being dispensed. In the continuous-flow mode liquid is pumped at the selected flow-rate until a stop signal is given. The display will show the actual volume in ml as it is dispensed. Self-calibration is a simple three-step process utilizing the LED display; once calibrated for the fitted tubing, any dose volume may be selected.

*For further information or a demonstration contact V. A. Howe & Company Ltd, 12–14 St. Ann's Crescent, London SW18 2LS. Tel.: 01 874 0422.*

**Circle No. 79 on Reader Enquiry Card**

### Stainer

Varistain 24-3, the latest in Shandon's line of automatic stainers, incorporates a 'Pass' function which allows any one or number of stations at any position in the staining routine to be bypassed. This facility is ideal where, for example, the operator wants to program and run two staining routines sharing common reagents, on a regular basis. By programming the stainer with 'Pass' at the relevant stations, the reagents specific to each routine can be selected automatically.

Another feature of the new stainer is a water-wash system, which provides a significant increase in flow-through capacity—up to 750 ml/min—without risk of blockage or overflow. This is made possible by a continuous circular channel, running behind the troughs. Water flows over a weir built into the back of each trough and into the channel at a rate which more than compensates for the

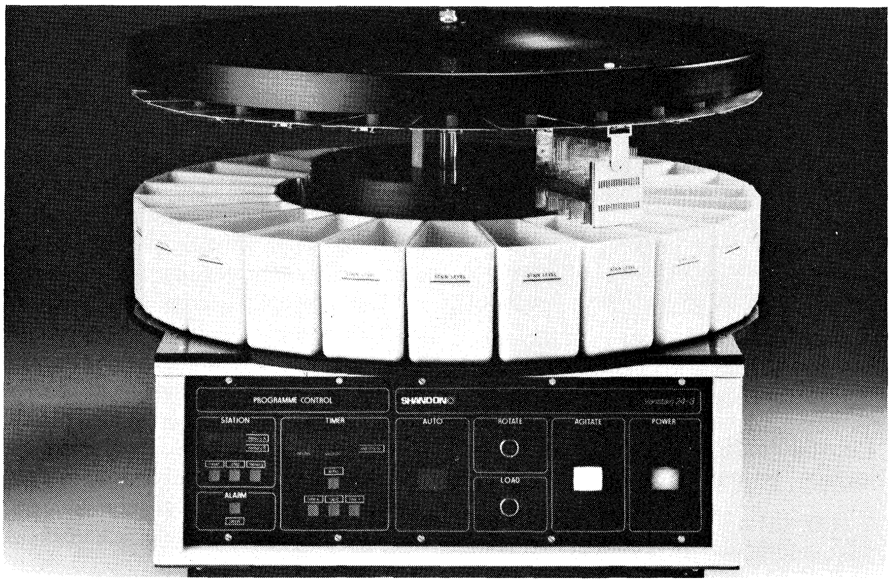

*Shandon's 24-position automatic stainer which was recently improved with all-electronic digital operating and programming functions and which has now further controls and other new features.*

displacement of water, caused by the descending slide carrier.

Three capacities of stainless-steel slide carrier are available—10 slides, 42 slides and 64 slides, they can all be used at any station to vary specimen throughput. The 42-slide carrier is adapted for use with the Autoslip (an automatic coverslipper), enabling slides to be transferred directly from staining to coverspilling, without being handled individually.

Using the Varistain 24-3 electronic controls and programming facilities, the operator can respond instantly to any changes in staining—variations in reagent strength for example.

Individual immersion times can also be reprogrammed to take account of changing conditions, without affecting the remainder of the program. Using the electronic timer and controls, the operator can program immersion times to the second in 1 s steps, up to 59 min and 59 s for every station.

Two independent program memories are incorporated, which hold the instructions for up to eight complete staining routines. And a new low-profile, extra-strengthened canopy is incorporated in the machine—this ensures reliability and prevents any misalignment of slide carriers as they descend into their staining troughs. When required, the canopy can be raised or lowered manually to provide added flexibility.

*Literature and specifications from: Shandon Southern Products Ltd. Chadwick Road, Astmoor, Runcorn, Cheshire WA7 1PR, UK. Tel.: 09285 66611.*

**Circle No. 80 on Reader Enquiry Card**

### Bio-Rad Laboratories

The new Bio-Rad Chemical Division Laboratories Catalogue contains information on various techniques, equipment and reagents in chromatography, electrophoresis, immunology and HPLC. Large sections have been added to the Catalogue this year covering HPLC equipment and consumables, immunoassay, immunoblot techniques, DNA sequencing and ion chromatography.

*For further information contact David Walton, Bio-Rad Laboratories Ltd, Caxton Way, Holywell Industrial Estate, Watford, Hertfordshire WD1 8RP, UK.*

**Circle No. 81 on Reader Enquiry Card**

### pH sensor

Solid-state reference electrodes are incorporated in TBI's pH sensors (these are available in Britain from Auriema Ltd). The design employs hardwood or permeable Teflon junction materials which give infinitely 'unpluggable' capillary characteristics. The whole of the reference is permanently charged with potassium chloride solution and the silver/silver chloride element is a remote member in the cell. A very low reference resistance is maintained, comparable to that of liquid types.

These solid-state pH sensors have been proven over long periods in arduous stream environments; a series of standard models offering a variety of construction types to suit process-application requirements has just been introduced. These include such variants as chemically-resistant body materials in Epoxy, Kynar and 316 stainless-steel, with features to suit in-line or submersible insertion and designs for facilitating removal from in-line systems without interruption.

A choice of electrode types is available with each of the TBI sensor models to give optimum performance for defined pH spans, temperature spans and chemically aggressive conditions. Rugged glass is used in the bulb area where vibration, rough handling and abrasive process streams are involved.

Hardwood liquid junctions are employed for most process streams except those including wood delignifers such as strong caustic and oxidizers (permeable Teflon is recommended for these cases).

TBI also produce a full range of associated electronics: locally mounted indicating transmitters and two-wire transmitters for example.

*Auriema Ltd are based at 442 Bath Road, Slough SL1 6BB, UK. Tel.: 06286 4353.*

**Circle No. 82 on Reader Enquiry Card**

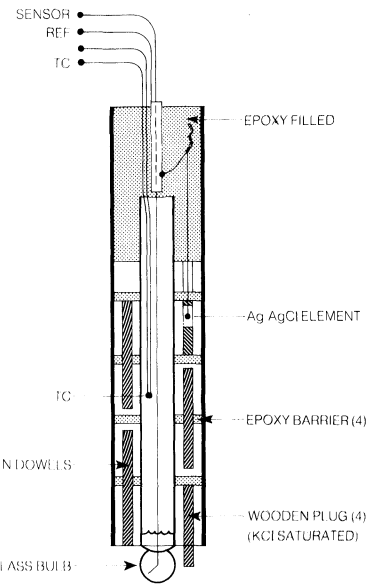

**The TBI Solid State pH Electrode**

*A novel design of pH sensors has brought long, stable service life and sustained accuracy previously thought impossible in the field of pH process measurement. TBI sensors are available in the UK from Auriema Ltd.*

### ICI micro software

Britain's largest chemical company ICI (Imperial Chemical Industries) has entered the world of microcomputer software with three packages aimed chiefly at professional scientists in industry, research and education. All are in regular use in ICI's own research laboratories. They are now offered externally under the

'Rexagan' trade-name already associated with the company's input/output interfaces for microcomputers.

The three packages are: Rexagan TOMULT, a multitasking programming system for writing complex real-time control applications in an extended version of BASIC; RSP, the Rexagan Statistical Package, for processing and evaluating arrays of experimental data; and Rexagan DROPTEST for assessing impact tests on materials.

First versions of the software are for the Commodore PET, with versions for other machines, including the Apple series and the Commodore 64, in preparation.

The software is to be marketed in Britain through distributors in the technical computer field, ICI are looking for similar agents in other countries.

*Details from Physics & Radioisotope Services, Imperial Chemical Industries PLC, PO Box 1, Billingham, Cleveland TS23 1LB, UK. Tel.: 0642 553661.*

**Circle No. 83 on Reader Enquiry Card**

### Life science TEM

Philips's EM 410 dedicated life science transmission electron microscope has been redesigned to increase throughput and efficiency. The new EM 410 LS is intended for users needing an instrument routinely capable of producing a large volume of high-contrast electron micrographs.

A novel column design reduces the machine's previous minimum magnification of 50× to 12× (for the initial overall survey of specimens). Ease of use is aided by a reduction of the point of magnification-range switch-over to around 500×, and an increase of maximum magnification from 310 000× to 500 000×.

The introduction of a normalization control, coupled with an easily reproducible specimen position, guarantees accurate repeatability of magnification over a long period of time—so comparisons of standards can be carried out with confidence.

High-fidelity imaging is achievable on a variety of specimen types: even at high tilt angles and with low-contrast specimens. This is due to a $\pm 60°$ eucentric goniometer stage, a range of accelerating voltages, the high-coherence Philips mini-gun and a high-contrast objective lens in the software-controlled optical column. High-quality imaging of thick specimens is possible, and extraction and optimization of information is aided by the ease with which stereo pairs can be produced using the eucentric stage. It is possible for the user to specify either the standard tungsten source or an $LaB_6$ high-brightness option, which is advantageous for microanalysis.

The clean, dry, differentially pumped vacuum system of the EM 400 is standard in the EM 410 LS.

A number of specimen holders is available for use with the EM 410 LS, including the Philips cryo-transfer system for frozen tissue.

Two dedicated lens programs ('BIOPROMS') are incorporated into the electron optics system, and are selectable from the operating panel. One is optimized to provide focus tracking from high to low magnifications and is a benefit in low-dose imaging of specimens, as well as in routine observation. The second is optimized for the stereo-imaging of thick specimens, through control of location of tilt axis in the image and virtual elimination of image rotation.

A simple five-stage sequence makes operation easy by carrying the user quickly through from specimen loading to foolproof, automated recording of the micrograph.

*Further information from Pye Unicam Ltd, York Street, Cambridge CB1 2PX, UK. Tel.: 0223 358866.*

**Circle No. 84 on Reader Enquiry Card**

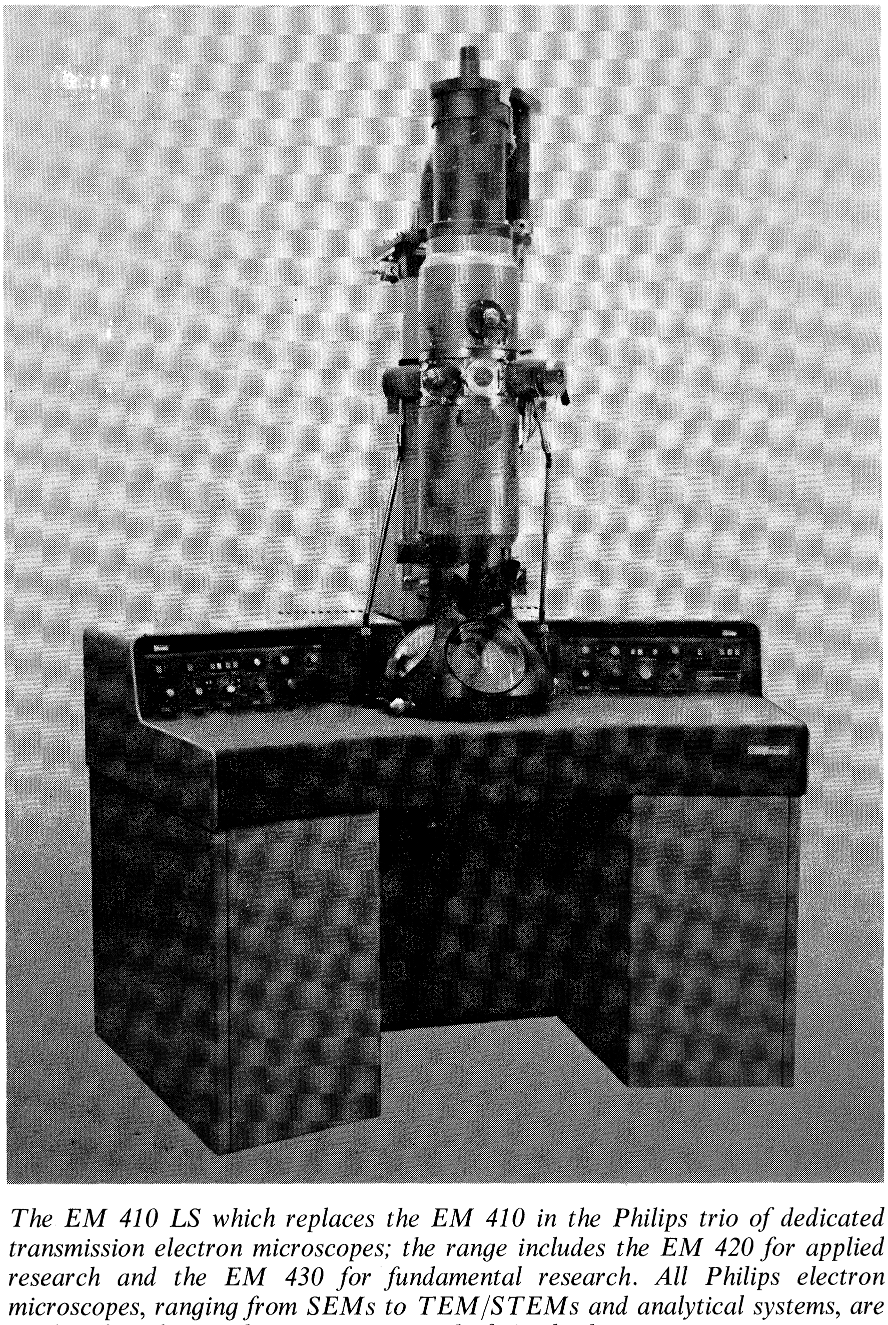

*The EM 410 LS which replaces the EM 410 in the Philips trio of dedicated transmission electron microscopes; the range includes the EM 420 for applied research and the EM 430 for fundamental research. All Philips electron microscopes, ranging from SEMs to TEM/STEMs and analytical systems, are marketed in the UK by Pye Unicam Ltd of Cambridge.*

### Photo diode array for HPLC

The SPD-M1A provides a 3D spectrochromatogram across the complete UV/Vis range and will enhance the performance of any HPLC. The real-time 3D plot gives instant identification of any interesting peaks and will show minor impurities in either sample or mobile phase which might otherwise be missed. Shimadzu's detector uses two types of lamp—deuterium to cover 200–380 nm and tungsten for 381 – 699 nm. This means, apparently, a sophisticated detection system and, because there are 512 diodes set only 25 μm apart, a sensitive response with high resolution is produced.

Three main detection ranges are available on this 'user-friendly' system. Firstly, single-wavelength monitoring with an added peak purity monitor; secondly, dual-wavelength range monitoring, comparing spectra obtained for components at two different wavelength ranges; and thirdly, the real-time 3D trace which produces a complete spectra from 200–699 nm, or any range in between. The SPD-M1A will store a complete spectra of any components from 200–699 nm, even though the user may require only a particular range to be plotted on the XY-T recorder. Any spectra can be looked at and parameters can be 'set' on the video screen before obtaining hard copy on the XY-T recorder. Spectra for particular components can be stored for future reference: for example, standard and sample spectra can be compared against any other peak or standard spectra for easy identification. The spectra will be automatically normalized for accurate identification. Shimadzu have incorporated 'data protection' in the machine—it can be left switched off for a month before data are lost.

*The SPD-M1A is being distributed in the UK by Dyson Instruments Ltd: Sunderland House, Station Road, Hetton, Houghton-le-Spring, Tyne and Wear DH5 0AT. Tel.: 0783 260452.*

**Circle No. 85 on Reader Enquiry Card**

### DMS for LIMS

A Data Management System (DMS) software package has been introduced by Varian for the VAX-11 Laboratory Information Management System (LIMS) that the company is jointly developing with Digital Equipment Corporation. The package is fully compatible with Digital's 32-bit minis and provides a centralized data-base management system for organizing data collection, testing samples and handling results.

Three concepts in the DMS software qualify the VAX-11 LIMS as a second-generation system. First, Varian's Instrument Network Architecture (INA) provides on-line data collection from a range of laboratory microcomputer devices: intelligent instruments, data systems, personal computers, and workstations. INA is based on the seven-layer International Standards Organization model for local area networks and supports protocols such as RS-232 and ETHERNET. In addition to collecting data, DMS schedules tests at workcentres, which may be instruments, locations, or persons. Secondly, the centralized data-base provides a unifying element to the laboratory: the data-base follows the principles set forth in the 1981 report of the Conference on Data System Languages (CODASYL) and ensures data integrity and security through access control. Thirdly, the system's modular architecture involves a workbench with software tools and interconnections. As a result, the VAX-11 LIMS can be easily

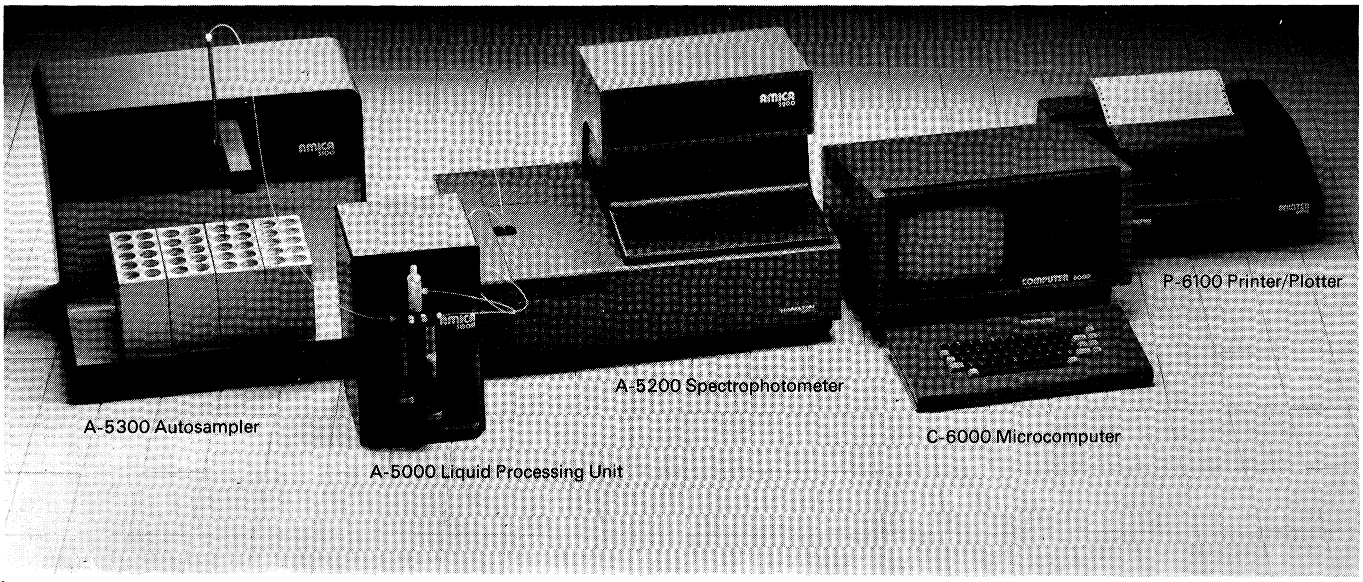

**AMICA — Automated Modules for Industrial Control Analysis**

AMICA systems are proving their efficiency, accuracy & reproducibility for laboratories where numerous samples are assayed using a number of methodologies.

TOTAL AUTOMATION from sample introduction to result documentation using:

**UV/VIS PHOTOMETRY, POTENTIOMETRIC OR PHOTOMETRIC TITRATION** *means*

**SPEED — ACCURACY — RELIABILITY**

We can also offer a Confidential Applications Service together with a 60+ Applications Bank of methods already documented — *so your Q.C. problem may already have been solved.*

Exclusive distributor for the UK:
V.A. Howe & Co. Ltd.
12-14 St.Ann's Crescent
London SW 18 2LS
Telephone (01) 874 0422 — Telex 262110

HAMILTON

**Circle No. 86 on Reader Enquiry Card**

modified with respect to size, diversity of instruments, types of applications, and degree of automation encountered in the laboratory. DMS also contains the interface to the sample management system module being developed by Digital: this will provide extended sample tracking and status information as samples move through the laboratory. Other modules will also be available—Varian's laboratory management system and packages from third-party manufacturers. The package is priced from $18 000 (depending on the configuration and size of the VAX-11 system). Delivery is scheduled for July 1984.

*Literature from Data Systems Operations, Varian Instruments Group, 2700 Mitchell Street, Walnut Creek, California 94598, USA. Tel.: 415 939 2400.*

Circle No. 87 on Reader Enquiry Card

### Low-cost GCs

A series of gas chromatographs, the 4300s, has been designed to operate with the latest Carlo Erba automation and data systems and will accept the full range of injectors and detectors. The basic chromatograph includes an analytical oven with base bodies for ionization and/or thermal conductivity detectors. The oven top is designed to accept two vapourizing injectors for packed or capillary (split/splitless) operation. Metal, glass or fused silica capillary columns can be used with the oven and they can be accessed by any combination of packed and/or split/splitless injectors. In turn, the injectors may be automated for up to 60 samples. The oven is controlled by an independent proportional temperature-controller and is prearranged for isothermal and temperature programming with a single-ramp multi-cycle programmer: an automatic cooling cycle is included at the end of each program. An optional cryogenic unit is also available, allowing subambient operation down to a maximum of −50°C. The oven has sufficient internal space for any additional valves needed for external columns, heart cutting, back-flushing and storing.

The 4300 retains full capabilities for coupled (parallel) and stacked (series) detectors: it is possible, for example, to couple FID, ECD and NSPD detectors to one column.

Auto sampling capabilities are based on the ASV 570, capable of holding 60 0·8 ml sample vials or 42 of 1·5 ml capacity. It is mounted on two rails spanning the width of the instrument, allowing a quick change from one injector to another and quick access for 'one-off' analyses that may be required during an automatic run. It is operable in local or remote mode, permitting simple integration with the instrument's base unit, temperature programmer or with an external integrator or computer. In all configurations, base parameters (analysis time, flush time) are variable between 1–99 min and seconds respectively.

The 4300 GCs can be used with many of the data systems developed for the Mega Series of chromatographs. The Mega Series integrators can act as an interface between the 4300 and the Carlo Erba HEC 960 computer—this allows a complete automatic injection, data-processing, gas-chromatograph system to be compiled for less than £12 000 (February 1984).

*Full technical information from Erba Science (UK) Ltd, Headlands Trading Estate, Swindon, Wiltshire SN2 6JQ. Tel.: 0793 33551.*

Circle No. 88 on Reader Enquiry Card

IMS 885 Automatic Ion Analyser System

RADIOMETER COPENHAGEN

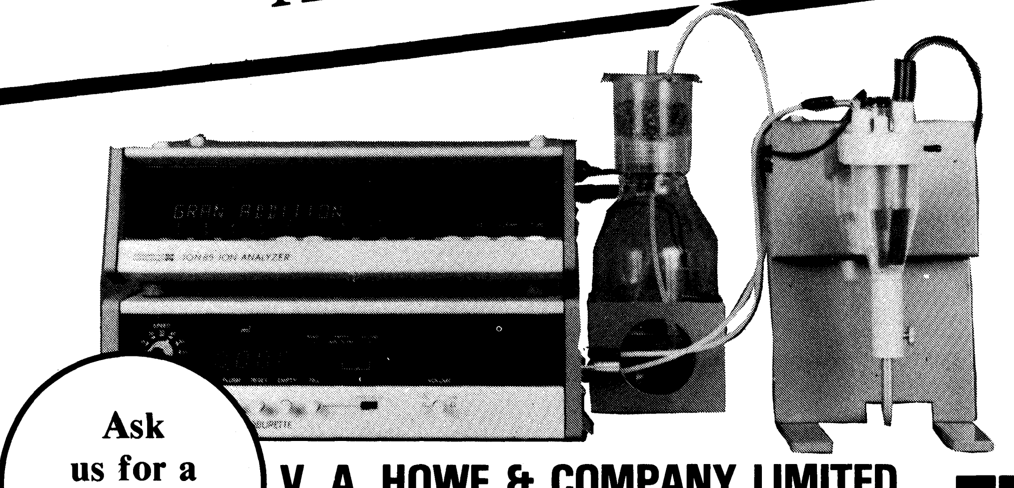

IMS 885 Gives you

- Automatic Reagent Addition for
  Grans Plot Titration
  Standard Addition/Subtraction
  Analate Addition/Subtraction
- Automatic Calculation of
  Detection Limit
  Electrode Slope
  Results In All Units
- Measurement of
  mV, pH, pX, Temp.
  and concentrates

Ask us for a demonstration

V. A. HOWE & COMPANY LIMITED

HOWE

12-14 ST. ANN'S CRESCENT,

LONDON SW18 2LS TEL 01-874 0422

Circle No. 89 on Reader Enquiry Card

### Atomic absorption spectrophotometers

Two new instruments in a series of atomic absorption spectrophotometers were announced at the end of last year by Allied Analytic Systems; the company was formed as a result of a merger of Instrumentation Laboratory's Analytical Division and Jarrell-Ash.

The IL *S-11* is a single-channel, single-beam system, and the IL *S-12* is a single-channel, double-beam system. The S-11 and S-12 are distinguished by three major features: (1) they use a high resolution (to 0·04 nm) monochromator similar to that in top-of-the-line instruments; (2) they are each upgradeable to a video-display instrument at a modest cost; and (3) they produce nearly perfect background correction by means of the Smith-Hieftje system. In the Smith-Hieftje system, the elemental hollow cathode lamp serves as its own background correction. When the lamp is operated at a low current, its light is absorbed both by the sample element (atomic absorption) and by the background. When a brief high-current pulse is passed through the lamp, atomic absorption is greatly reduced, but background absorption remains the same. When the high-current result is subtracted from the low-current result, background correction has been achieved. Because the same light source is used for atomic absorption and for background correction, misalignment is impossible. The system can correct for background absorption of up to 99·9%; it can also compensate for 'structured' background and for some spectral interferences.

Additional features of the S-11 and S-12 include data read-out on a four-digit LED based on individual readings or running mean; analytical working curves can be generated with a blank and up to five standards; a burner system with comprehensive safety features; a simplified, user-friendly and spill-proof keyboard with 14 function keys; and a three-lamp holder for convenient storage of hollow cathode lamps.

Optional features and equipment for the S-11 and S-12 are an automatic wavelength drive; a four-lamp turret; and inkless printer; RS232C interface computer memory protection; and a deuterium arc background corrector to complement the Smith-Hieftje system.

*Further information from Instrumentation Laboratory, One Burtt Road, Andover, Massachusetts 01810, USA; or Jarrell-Ash, 590 Lincoln Street, Waltham, Massachusetts 02254, USA.*

**Circle No. 90 on Reader Enquiry Card**

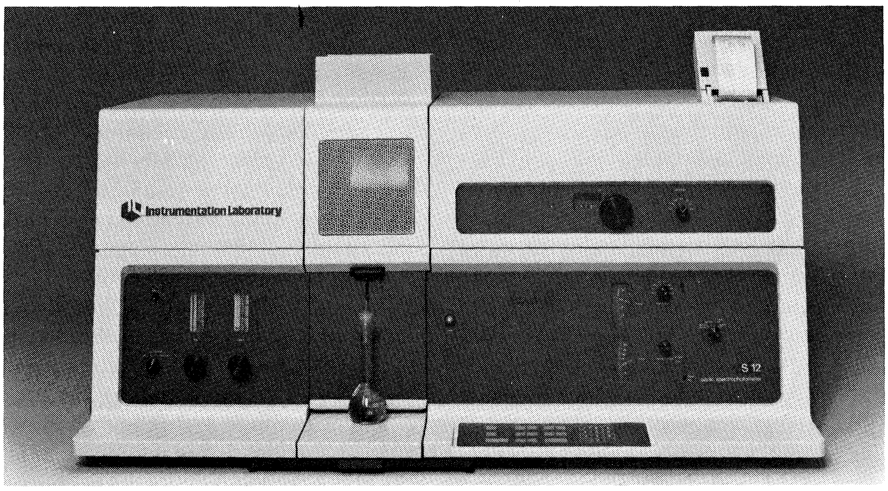

*The S-12: a single-channel, double-beam atomic absorption spectrophotometer. The machine uses the Smith-Hieftje system which is of particular value in the analysis of biochemical samples, seawater and metal alloys.*

### Cross assemblers

The Sira range of low-cost assemblers to support the commonly-used processors has been extended still further, so that it now includes:

Intel 8080/5, 8048, 8041, 8021, 8022
Motorola 6800, 6801, 6802, 6803, 6805, 6808, 6809
RCA 1802, 1804, 1805, 1806
Zilog Z80
National NSC800, SC/MP
Mostek 6502.

These cross assemblers are designed to run on DEC machines under RT-11 or RSX-11M. They are priced at around £500 for one processor plus *c.*£250 for each additional processor purchased at the same time. Source files for the cross assemblers are written using the processor manufacturer's standard mnemonic codes. Up to three output files can be generated: an object file, a list file, and a PROM file. The object file can be generated in the device manufacturer's standard HEX format, in Tektronix standard HEX format compatible with the 8001 Development Laboratory, or as a binary file. The list file combines the original source file with the object file. The PROM file is in HEX space format suitable for most commercial PROM programs. A checksum facility is available in the assembler to permit continuous run-time checking of program memory in the target system. Full installation and operating instructions are included in the package.

*Technical enquiries to Eric Goodyear, Sira Ltd, South Hill, Chiselhurst, Kent BR7 5EH, UK. Tel.: 01 467 2636.*

**Circle No. 91 on Reader Enquiry Card**

### Pye Unicam

Ten instruments are now offered by Pye Unicam for infra-red spectroscopy. The SP3 'S' series is the second recent addition to the company's range; the four machines in the series are simple to use and include the Spectraset system which automatically optimizes instrument operating parameters according to the type of sample being analysed.

*More information from Pye Unicam Ltd, York Street, Cambridge CB1 2PX, UK. Tel.: 0223 358866.*

**Circle No. 92 on Reader Enquiry Card**

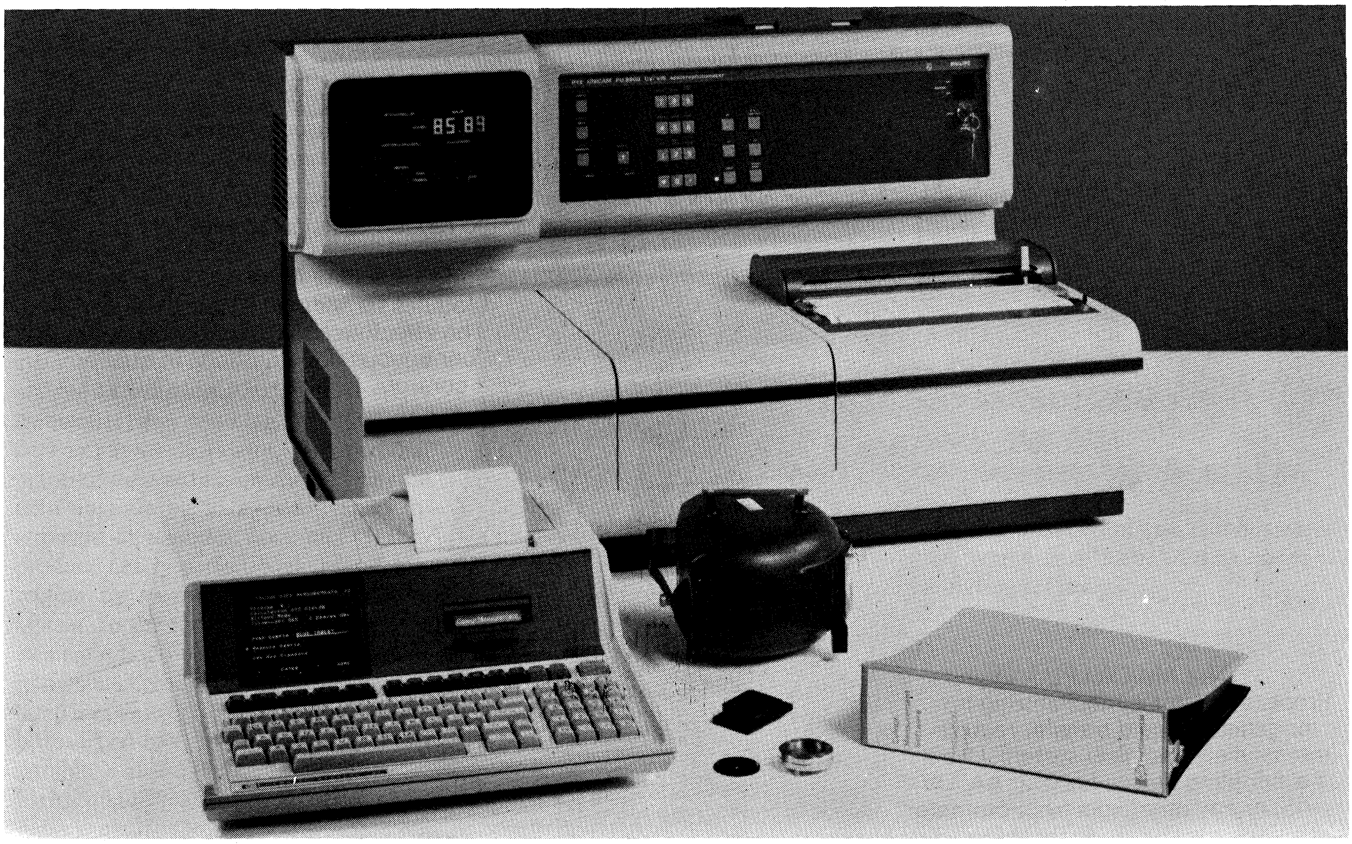

*Pye Unicam's new colour-measurement system, which is based on the PU 8800 UV/V is spectrophotometer. The new system employs an integrating spheroid and a fast, powerful software package for the HP 85 computer. It is the only non-dedicated system that completely conforms to the requirements of the CIE, the governing body of colour science. Details from Pye Unicam Ltd, York Street, Cambridge CB1 2PX, UK. Tel.: 0223 358866.*

**Circle No. 94 on Reader Enquiry Card**

### Twin-bed demineralizer

Feedwater's Deminpac is compact, simple to operate, fully skid-mounted and produces distilled water economically. It is available in a variety of sizes and sold as a complete package with all ancillary equipment. Applications include rinse water for circuit-board cleaning, electroplating and metal parts prior to painting, process water for paint and chemical manufacturers, film processing, textile manufacturing and printing and clean-up water in laboratories. Normal commercial strength regenerant chemicals are used; the chemicals are drawn direct from supply carboys and resin regeneration is initiated either automatically or manually. Any cold, potable, colourless water free from suspended solids and excessive organic contamination is suitable for purification by the Deminpac.

Treated water quality is, of course, dependent on the feedwater, but with raw water of 350 ppm total dissolved solids the instrument will produce a purified water with 2 ppm total dissolved solids.

*Details from Feedwater Services Ltd, Arrowebrook House, 2 The Grove, Poulton Road, Wallasey, Merseyside, UK Tel.: 051 630 3373.*

**Circle No. 95 on Reader Enquiry Card**

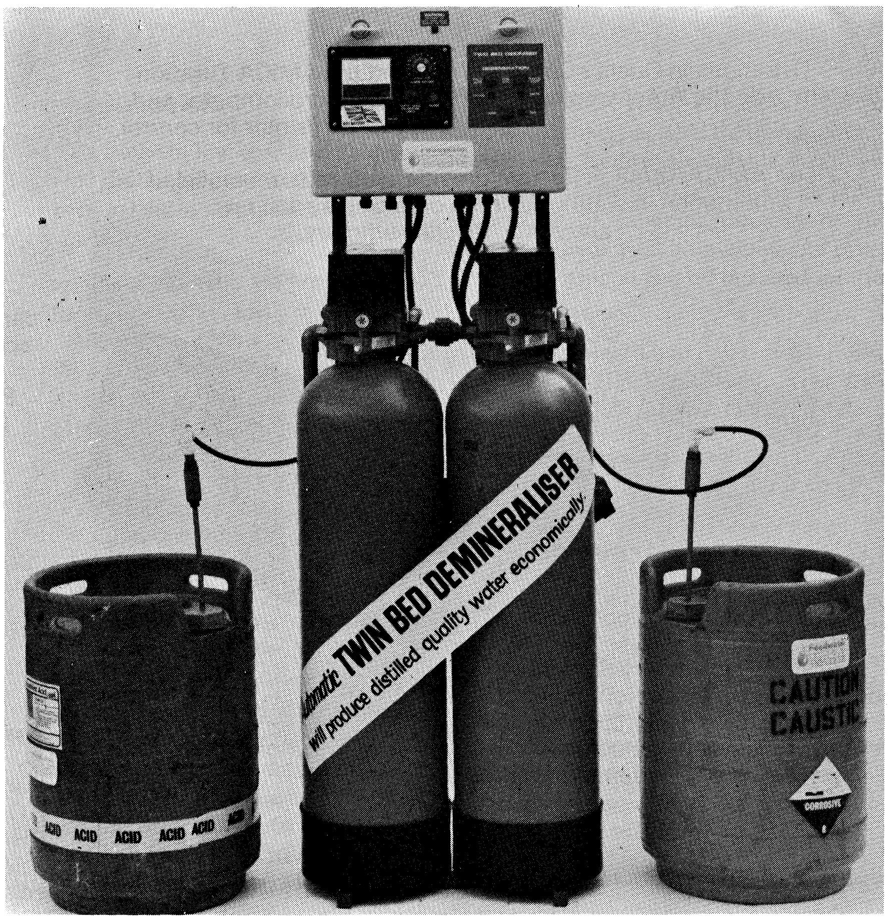

*'We have responded to the need for an advanced design of twin-bed demineralizer with a more simplified and compact control module'. (Pat Revans, Feedwater.)*

### Full range HPLC pump

Kratos's Spectroflow 400 solvent delivery system is described as offering high reliability, high accuracy, and broad flow rate range at a low cost. The instrument's flow range is 10 μl/min to 5 ml/min, which makes it suitable for microbore LC, fast LC, and conventional HPLC. Unlike many other pumps, it is not necessary to change pump heads or electronics to operate at microbore flow rates. Accuracy is $\pm 0{\cdot}1\%$ of setting, made possible by the pump's unique miniaturized check valve design, and quartz frequency synchronized pump drive.

The Spectroflow 400 also includes remote control provisions, suiting it for automated use under the control of virtually any systems controller or computer, usually without the need for an interface.

It can be operated at pressures up to 7000 psi; front-panel controls permit user selection of upper and lower pressure limits and system purging capability.

In order to help minimize maintenance the Spectroflow 400 dual piston design uses only one pair of check valves.

*The price is $3600 (USA), availability beginning September 1983. Details from Kratos Analytical Instruments, 170 Williams Drive, Ramsey, New Jersey 07446, USA.*

**Circle No. 96 on Reader Enquiry Card**

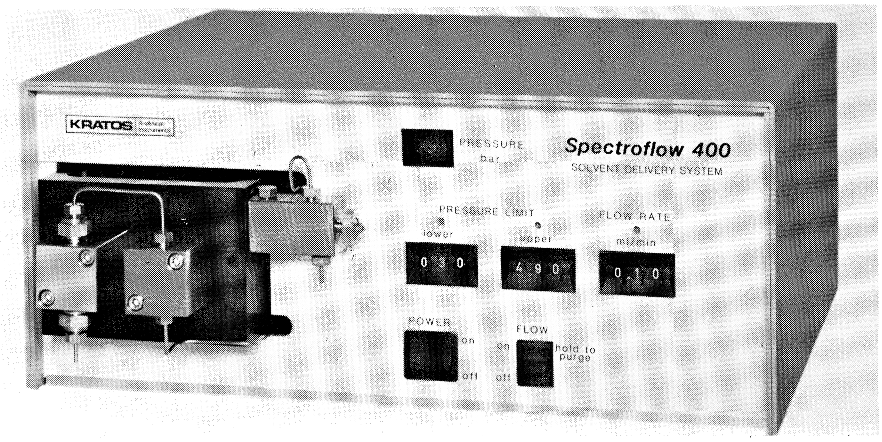

*As part of Kratos s introductory programme the Spectroflow 400 solvent delivery system is being sold with a year's service extension on the standard warranty. The manufacturer claims increased operational reliability over previous and competing models.*

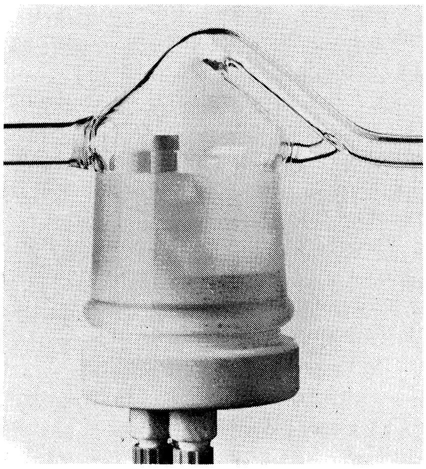

*The Stop-Flow GMK nebulizer for the Labtam Plasmascan 710 ICP atomic emission spectrophotometer, which is being distributed in the UK by Techmation. It incorporates changes which overcome problems associated with maintenance and features a new operating system eliminating short-term instability and long-term drift, reducing memory effects and shortening analysis time. Information from Techmation (58 Edgware Way, Edgware, Middlesex HA8 8JP, UK).*

**Circle No. 97 on Reader Enquiry Card**

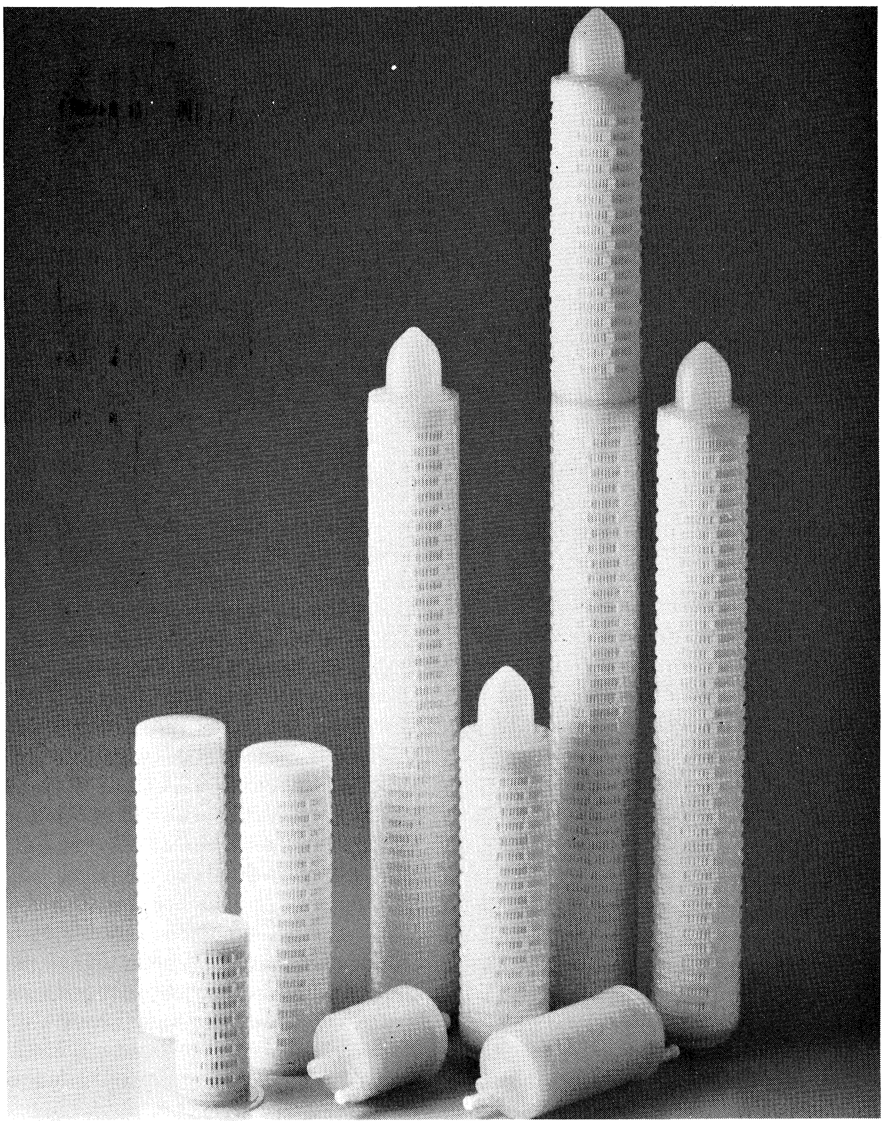

*Sartorius's range of high-quality chemically resistant filter cartridges. There are two main product lines: prefilters (Sartopure) made from polypropylene and available in sizes varying from 50 μm to 0·2 μm for use in filtration of solvents and acids. And PTFE filter cartridges (Sartofluor) with 0·2 μm and 0·45 μm membranes. Both products are well suited to work with solvents and acids in the electronics industry, solvent recovery, the cleaning of Freon and the filtration of solvents in the pharmaceutical industry. Sartorius Instruments Ltd are based at 18 Avenue Road, Belmont, Surrey, UK.*

**Circle No. 98 on Reader Enquiry Card**

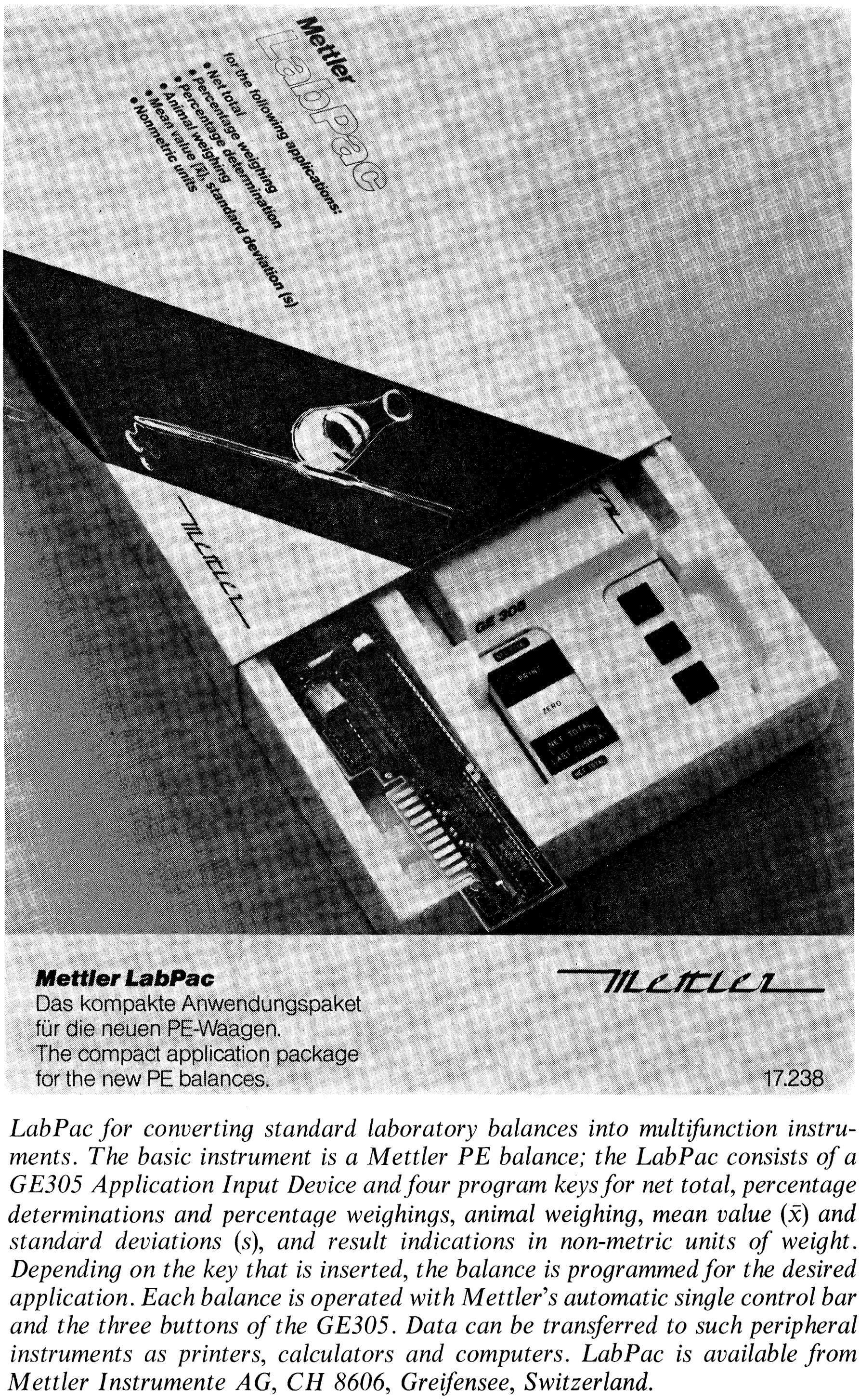

*LabPac for converting standard laboratory balances into multifunction instruments. The basic instrument is a Mettler PE balance; the LabPac consists of a GE305 Application Input Device and four program keys for net total, percentage determinations and percentage weighings, animal weighing, mean value (x̄) and standard deviations (s), and result indications in non-metric units of weight. Depending on the key that is inserted, the balance is programmed for the desired application. Each balance is operated with Mettler's automatic single control bar and the three buttons of the GE305. Data can be transferred to such peripheral instruments as printers, calculators and computers. LabPac is available from Mettler Instrumente AG, CH 8606, Greifensee, Switzerland.*

Circle No. 99 on Reader Enquiry Card

### FIA system

Advanced Medical Supplies and the Laboratory of the Government Chemist are now marketing a joint-venture flow-injection analysis system. The system took two years to develop and it is likely to have a considerable impact because it will generally cost only a fraction of the price of competitive FIA systems.

In operation, a sample is pumped from a sample cup through a fixed-length sample loop of PTFE tubing connected to a chemically-inert eight-port valve. On receipt of a 'sample injection' signal, the content of this sample loop is transferred by the valve into a continuously-flowing carrier stream. At fixed intervals, determined by their distance from the sampling valve, reagent or reagents are added to the main stream. This combined stream is passed through a coil of PTFE tubing, which acts as a delay/heating coil for colour development and then into a flow cell where the colour intensity is measured by an integral colorimeter. The measured intensity is displayed on a liquid-crystal digital panel meter and can be output to a potentiometric recorder.

The range of methods available cover the following analytes: chloride, phosphate, nitrate, nitrite, ammonia, free chlorine, iron, sulphate, anionic detergents, pH and conductivity.

The analyser is normally supplied already set up and optimized for a specific analysis. Standard reagent packs are available.

*Technical specification from Advanced Medical Supplies Ltd, 19 Holder Road, North Lane Industrial Estate, Aldershot, Hampshire, UK.*

Circle No. 100 on Reader Enquiry Card

### Fourth-generation continuous-flow analysis systems

System 4 is a new analytical system for the user who wants to perform single or multiple analyses of a sample. It is modular so that the user has the flexibility to tailor the analytical system—it can be readily altered to a different series of chemistries or expanded to meet new demands by the addition of further modules. Upgrading a simple manual system to a fully automated analyser can be achieved in stages as finances allow.

System 4 is described as fast, accurate and precise, with economy, efficiency and simplicity as the key features. Samples are processed at a rate of up to 120 samples/h, with simultaneous analysis on all chemistry channels. It is easy to operate and requires no operator intervention between initiating sampling and collecting the results.

A range of chemistries is available: agriculture, clinical, biochemical, food, industrial and water analyses are examples of the applications for which System 4 may be used.

System 4 employs continuous-flow analysis. In the past, this technique has been the subject of considerable criticism on the grounds of:

Slow sampling speed
Analysis by peak height on recorder trace
Wasteful on reagents
Long warm-up time
Long time to wash through
Occupies too much bench space
Lack of precision due to carry-over problems
Not easy to run urgent samples
Large sample volume required
Generally untidy appearance.

The System 4 sampling rate is considerably better than the 40–60 samples of previous continuous-flow analysers. This improvement in the sampling rate has been achieved by incorporating a new design of sampling head, refining the layout of the chemistry cartridges and

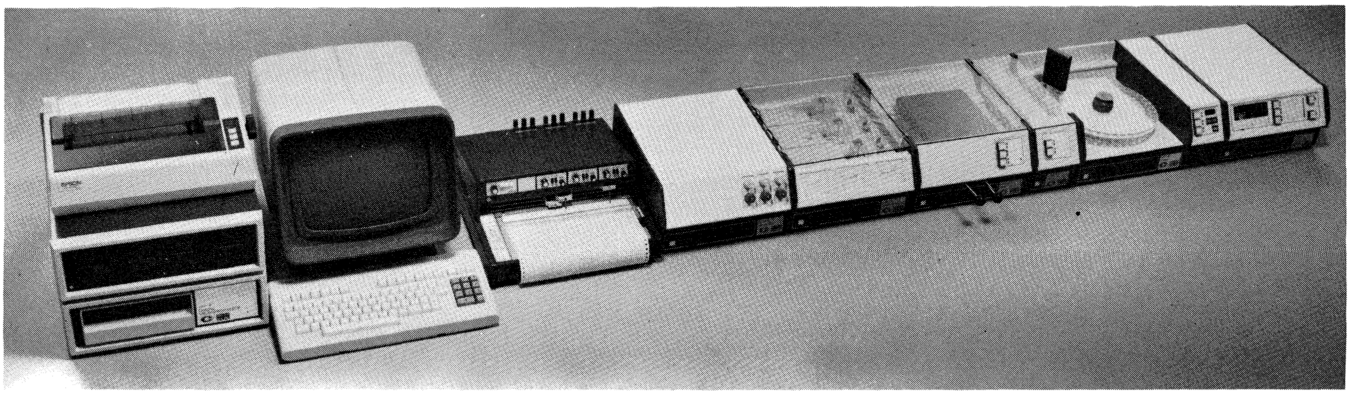

*System 4 from ChemLab Instruments Ltd. The continuous-flow system has the following features: 'simplicity, flexibility, multiple analysis, high throughput, high capacity, precision, accuracy, reliability, low reagent cost, small sample volume, urgent samples and stand-by facilities, time control, data-processing and ergonomic design'.*

miniaturization of the colorimetry. The effect of these changes is a reduction in the carry-over, with consequent improvement in precision, and a reduction in the reagent consumption and sample volume requirements. The analyser uses standard laboratory reagents, rather than expensive specialized kits. Further savings in reagents are achieved by an automatic stand-by facility which comes into operation on completion of the run. This facility is only available with the 'control module'.

Incorporating a control module into a system provides an automatic warm-up and shut-down capability. At the end of a day the analyser can be programmed to complete a batch of samples, wash through and close down the system without operator involvement; the following morning the control module will switch on the analyser so that it is ready to run when the operator arrives.

Urgent samples may be processed immediately if a run is in progress, within 10 min if the system is in stand-by and within 30 min if the system is in shut-down. Special attention has been paid to the design of the whole system to minimize the lengths of flow-lines to improve performance and to eliminate the 'spaghetti-like' appearance normally associated with continuous-flow analysers.

The Dataflo microprocessor is a sophisticated data-analysis package designed to cater for the continuous-flow user. Up to 16 channels of data can be processed simultaneously, without the need for synchronization. Results are printed as concentration values with automatic calibration, drift and wash correction and sample identification. An additional feature of the Dataflo is a laboratory data management capability enabling the user to produce reports to any desired format and to store results.

*System 4's manufacturer*

ChemLab Instruments Ltd was founded in 1967 to supply scientific instruments to chemical, biochemical and medical laboratories. At that time it was felt there was a need for a company, which, by concentrating on specialist laboratory instrumentation, could offer a better service and technical back-up than the general laboratory equipment suppliers. Experience showed this to be the case and ChemLab was soon handling sophisticated laboratory instrumentation from both British and overseas manufacturers—in the latter case there were no British alternatives to the products being offered.

As business increased, frequent contact with research laboratories revealed there was a need for specialist instruments to cater for the new techniques being evolved in the rapidly developing fields of biochemistry, clinical chemistry and medical research.

In 1970 ChemLab began manufacturing a number of instruments based on ideas and suggestions made by laboratories. Initially they were produced in a home workshop on a part-time basis, but the demand quickly reached the level where it justified setting up a full-time manufacturing company.

In 1971 ChemLab Manufacturing Ltd was established in a small factory on the Rayleigh Weir Industrial Estate, Rayleigh, Essex, UK. The new facilities enabled the range of manufactured products to be increased and the company's marketing organization expanded to promote sales over a wider area.

One of the first and most important products to be developed was a range of instruments which comprised a system for performing automatic chemical analysis; these systems sold to hospitals, universities, technical colleges and industrial concerns throughout the UK.

The manufacturing capacity of the Rayleigh Weir factory soon proved unable to cope with the increasing home and export sales and the new products that were being introduced. It was therefore necessary to look for new, larger premises. A site was found on the Burnt Mills Industrial Estate, Basildon, Essex, and a new factory built. As the business grew, new departments were added until today the site houses the R & D Section, Development Laboratory, Design Office and Experimental Electronics Section, in addition to the normal manufacturing departments.

*Further information from ChemLab Instruments Ltd, 129 Upminster Road, Hornchurch, Essex RM11 3XJ, UK. Tel.: 04024 57011.*

**Circle No. 101 on Reader Enquiry Card**

### Sample-injection valve

FiAtron's FIA-valve/2000 is a programmable, all Teflon, sample-injection valve for flow-injection analysis, low pressure liquid chromatography and other laboratory solution-handling applications. The valve is a rotary, bidirectional arrangement with a fixed, 3 $\mu$l internal sample loop, an external, user-changeable sample loop and a programmable, time-based sampling mode. Each sampling loop or mode can be used independently. It has an RS-232 port for easy interfacing with external computers, a remote on/off switch, four user programmable TTL or relay switches and a dedicated output for controlling FiAtron's SC-110 sample changer.

*More from FiAtron Systems, Inc., 6651 North Sidney Place, Milwaukee, Wisconsin 53209, USA. Tel.: 414 351 6650.*

**Circle No. 102 on Reader Enquiry Card**

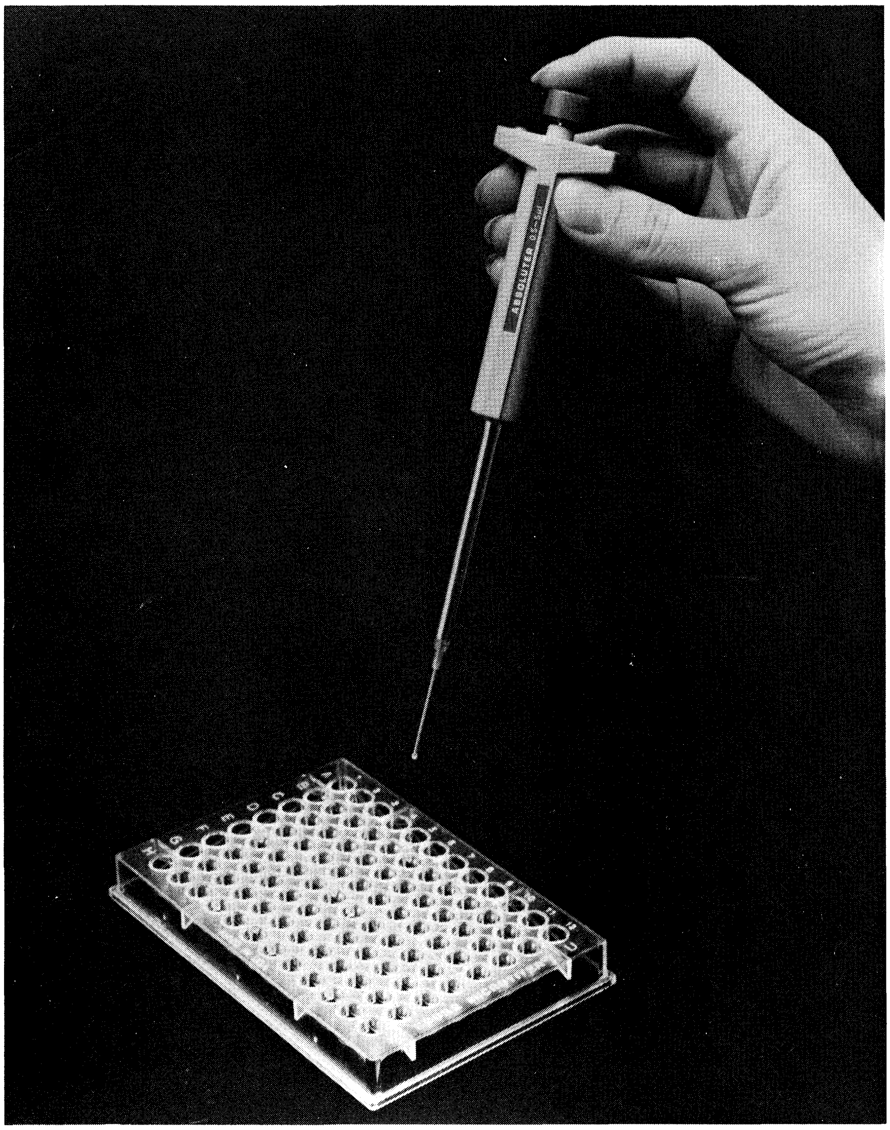

*'Ultra-Micro Absoluter': a soft-delivery positive displacement ultra-micro pipettor with Teflon tips for soft delivery, Tri-Continent Scientific's new product is intended for applications where high-pressure sample delivery should be avoided: RID and TLC for instance. It comes in two adjustable models: 5–5 μl and 2–10 μl, delivering samples with positive-displacement accuracy and at a low-pressure to maintain the integrity of gel surfaces and wells. Both models feature '30-second' in-lab calibration and smooth ultra-low actuation pressures. More information from either Tri-Continent Scientific, Inc., 12541 Loma Rica Drive, Grass Valley, California 95945, USA, or (in Europe) 1 chemin de Chandieu, 1006 Lausanne, Switzerland.*

**Circle No. 103 on Reader Enquiry Card**

### Research GC/MS

Features and specifications previously found in much higher priced quadrupole instruments are offered, according to Finnigan MAT, in the Model 5100 research GC/MS. It uses a data system called SuperIncos (designed especially for the instrument), which is described as providing optimized control of GC/MS operations and advanced application software. The data system was designed to efficiently handle large amounts of information from complex samples and it provides a good selection of output forms. The company say that the real significance of the SuperIncos software can be found in the details of the operating programs. As an example, a mass spectrometer control program allows the user to create simple descriptors to sequence the ionizer filament and electron multiplier on/off, change scan ranges, and switch between positive and negative ion detection during an experiment.

The 5100 system also uses the latest technologies: Finnigan MAT pioneered the application of local area networks (LAN) in GC/MS laboratories. A LAN permits multiple SuperIncos data systems to communicate with each other and a central laboratory computer. Also available in a separate optional computer system, the Mass Spectral Data Processor (MSDP), which interfaces via a serial communication PCB to an existing Incos data system. The MSDP is designed to overcome the bottleneck associated with the data-processing of large numbers of complex samples. A 35-megabyte Winchester-type disk drive is a standard feature of the system. It requires no head adjustments or alignments, and the medium operates in a completely sealed environment, which improves reliability and efficiency over disk drives with removable media. A 20-megabyte cartridge tape provides a removable medium for archival storage of data. And a new video terminal on the 5100 offers up-to-date keyboard and graphic display features. The detachable keyboard can be moved up to two feet from the terminal.

*A brochure describing the system's features and applications and a data sheet providing specifications is available from Finnigan MAT, 355 River Oaks Parkway, San Jose, California 95134, USA. Tel.: 408 946 4848.*

**Circle No. 104 on Reader Enquiry Card**

### 'Autozap'

Designed for testing ESD (electrostatic discharge) sensitivity in semiconductors, Hartley Measurements' Autozap-200 complements the company's range of high-voltage test systems for cables and passive components. It provides a fast, convenient and safe method of testing; semiconductors particularly at risk from ESD include microprocessors, memories, interface controllers, UARTS, as well as other medium and large-scale MOS devices. Autozap's discharge test circuit, which simulates the 'human-body' electrostatic discharge model, generates precise amounts of energy at high voltage and deposits them into the device on test under carefully controlled conditions. Since the complete test sequence for each device (which can have up to 40 pins) is carried out fully automatically, the error inherent in manual methods is totally avoided. It is fully controlled by computer. The control system implements the ESD test standards called up by STACK-001 and MIL-STD883·b. However, using commands given in plain English, it is also possible for the user to implement special test sequences without modification to any part of the system. Extensive self-testing and error-checking routines are included.

*Full details from Hartley Measurements Ltd, Unit 4, Bear Court, Basingstoke, Hampshire RG24 0QT, UK. Tel.: 0256 56695.*

**Circle No. 105 on Reader Enquiry Card**

### Monochromator

The THR1000 monochromator/spectrograph was developed for high-resolution spectral analysis. CAD techniques were used to calculate the best resolution versus luminosity compromise. The manufacturer's complete line of classically ruled, conventional and blazed holographic gratings allows the THR1000 to be used over a wide spectral range. Straight or curved slits are available, as well as adapters for photographic and multichannel detectors. The instrument is equipped with a stepping motor driven by the 'SPECTRALINK' system. Completely modular, the SPECTRALINK spectrometer drive and data acquisition system is the only one to couple directly both these functions in one package; it can either be used by itself or each of these functions can be directly addressed from a computer.

*Main features:*
- Focal length: 1 m
- Aperture: *f*/7·5 or *f*/9
- Grating size: 1120 × 140 mm or 110 × 110 mm
- Resolution: 0·008 nm at 500 nm with 1200 g/mm grating and reduced slits
- Dispersion: 0·8 nm/mm with 1200 g/mm grating
- Stray light rejection: $10^{-5}$ at 1 nm from 514·4 nm laser line.

*For more details and price information contact EDT Research, the sole UK representatives of Jobin-Yvon, at 1 Trading Estate Road, London NW10 7LU. Tel.: 01 961 1477.*

**Circle No. 106 on Reader Enquiry Card**

### Automatic sampler

Up to 50 sample vials can be handled by Shimadzu's AOC-9 pneumatically operated syringe-type automatic sampler. It was developed for use with the company's GC9A gas chromatograph and is intended to automate all sampling procedures, syringe washing, measurement of sample volume and injection.

All operations are controlled via the keyboard of the GC9A (or from a C-R2A integrator if required). The volume of sample to be injected is selectable in four steps of 1 μl, 2 μl, 4 μl and 8 μl and the same sample can be injected up to 10 times to ensure a higher reliability. In order that the most suitable solvent is used to wash the syringe, 50 solvent vials can be placed for 50 samples. Even viscous solution samples can be drawn into the syringe by adjusting suction time. Samples can be injected into either injection port.

When the GC9A is connected to the C-R2A data processor, the operational parameters of the sampler can be listed on the VDU or the printer/plotter and controlled by the user-defined BASIC program. Use of the C-R2A also allows the system to check analyses against expectation and, if necessary, repeat the analysis.

*The AOC-9 is being handled in the UK by Dyson Instruments Ltd: Sunderland House, Station Road, Hetton, Houghton-le-Spring, Tyne & Wear DH5 0AT, UK. Tel.: 0783 260452.*

**Circle No. 107 on Reader Enquiry Card**

### 'Personal' computers and titration

The D470 Titration Kit from Radiometer is a complete package connecting Radiometer titration equipment to a Hewlett-Packard HP-85 micro. The fully documented, standard tape is preprogrammed to simply perform computerized titrations: the tape is inserted into the HP-85 and only four soft keys have to be operated for routine titration. Equivalence points are automatically detected as the inflection points of the titration curve. The titration results and curve are displayed on the HP-85's screen and can be plotted by the built-in printer/plotter. Radiometer titration equipment is modular so a system can be configured to specific requirements.

*Further details from V. A. Howe & Co. Ltd, 12–14 St. Ann's Crescent, London SW18 2LS. Tel.: 01 874 0422.*

**Circle No. 108 on Reader Enquiry Card**

### Repetitive dispenser

Stat-Matic II is a light-weight and easily operated repetitive dispenser. It can dispense from an attached or a remote bottle and there are four models available, which cover a volume range from 10 μl to 2 ml. Accuracy is claimed to be ± 1% and reproducibility ± 1%G.V. A repriming tube and port permit a near-zero loss and internal volume-calibration rings allow fixed-volume reliability with the flexibility for changing volumes if necessary. The Stat-Matic includes six adapters to fit most bottles, including non-threaded vials. The dispenser is also virtually unbreakable: there is no exposed glass and plungers are either Teflon or Teflon-coated.

*Full details from Tri-Continent Scientific, Inc., 12541 Loma Rica Drive, Grass Valley, California 95945, USA. Tel.: 916 273 8389.*

**Circle No. 109 on Reader Enquiry Card**

### High-speed digital recorder

The Astro-Graph is a high-speed, high-resolution instrument which provides a unique combination of analogue writing, printing and graphic representation of digital or analogue data. It can perform functions which previously needed several machines: high-speed analogue recorder, X/Y recorder, printer and data logger.

The Astro-Graph features a microprocessor-driven linear-array thermal printhead to produce a dot density of eight dots/mm, adequate for high-resolution tracing, which in its trace, is completely free from digitizing or interpolation steps. RIL and the manufacturer, Astro-Med, believe the ability of Astro-Graph to produce a precise hard copy in a variety of data configurations promises significant improvements in the quality and usefulness of medical, telecommunications, aerospace and industrial process analysis. The use of a thermal printhead permits fast responses and disposes of the limitations imposed by the inertia of a mechanical stylus. The Astro-Graph provides faithful representation of high amplitudes at high frequencies in analogue tracing. A variety of standard software is available, along with self-generated grid patterns; a page printer format with an 80 character column printer; and a graphic format with individual dot address by 16 bit parallel input.

*Further information from RIL (Russet Instruments Ltd), Unit 1, Nimrod Industrial Estate, Reading, Berkshire RG2 0EB, UK.*

**Circle No. 110 on Reader Enquiry Card**

### Single-phase a.c. and d.c. motor valve actuators

Electric valve actuators powered by single-phase a.c. motors or d.c. motors are being offered by Rotork Controls. Previously the company's standard range has been three-phase a.c. only—there are two reasons for the development. The first is a growing requirement for motorization of more and smaller valves in remote areas, where the convenience and economy of being able to tap into existing single-phase supplies is a great advantage. And there is also a developing need, apparently, for an alternative to the 'one-shot' pneumatic fail-safe method of operating valves in the event of a failure of the electricity mains. A d.c. powered actuator, normally operating on a rectified mains supply, can be switched to a stand-by battery supply in such an emergency. Again, in the water- and waste-treatment industry, where plants may be unmanned or operate at low manning levels, or in the power industry where boilers may need rapid shut-down, a d.c. valve actuator can provide an efficient and relatively low-cost fail-safe mode. Single-phase a.c. actuators suitable for operation from 240 V supplies up to Rotork's size 11A (maximum torque 68 Nm at 18 rpm 50 Hz, 22 kN thrust) and d.c. up to size 16A (torque 203 Nm at 48 rpm, 67 kN thrust) for 48, 60, 110 or 220 V are now available. Further sizes in the single-phase range will be introduced soon.

*More information from Rotork Controls Ltd, Lower Weston, Bath, Avon, UK. Tel.: 0225 28451.*

**Circle No. 111 on Reader Enquiry Card**

### Turbidity recording

A complete system for continuous-flow turbidity readings, suitable for unattended use in remote locations, is available from Techmation. The system, supplied complete in weather-proof housing, comprises a Turner Designs Model 40 nephelometer fitted with a modular flow-cell and linked to a 30-day recorder. Stabilizing circuits in the nephelometer keep readings to within ±0·5%. Recorders fitted with a relay contact to turn other equipment on and off can be used, adding a useful control facility to the system.

*Details from Techmation Ltd, 58 Edgware Way, Edgware, Middlesex HA8 8JP, UK. Tel.: 01 958 3111.*

**Circle No. 112 on Reader Enquiry Card**

### Lingot sorting

The *in situ* sorting of materials has been an industrial problem for some years: being sure that a given steel lingot is not mixed with others of a different quality can be very important. Semi-finished products in storage areas are numerous and varied; their identification must be simple and rapid. This does not usually involve repeating a precise quantitative analysis, rather, the confirmation that the metal belongs to a predefined grade. The JY Q500 was designed to perform rapid *in situ* identification.

Mounted on four wheels, the JY Q500 spectrometer is air-tight and insensitive to atmospheric conditions. It can be easily moved from one storage area to another. Connected to the spectrometer by an optical fibre whose maximum length is 13 m, a gun is used to create a spark on the piece to analyse, without the necessity of removing it from its storage area. A microprocessor processes the signal and indicates whether or not the metal belongs to the grade considered.

The spectrometer uses the same optics as the JY 32 laboratory instrument. It has high resolving power at 5·5 Å/mm in first order and 2·75 Å/mm in second order. The polychromator is made of 'Ni-Resist' and leads to an easy choice of up to 24 elements among 110 prepositioned spectral lines. The instrument was designed to enable low concentrations of carbon to be assayed: ⩽0·10%C.

Six excitation conditions are available and preselected. This wide choice enables the instrument to be adapted to all types of metals.

Located in a sealed housing, the electronics and the microprocessor lead to a simple and rational utilization of the instrument. The user has the choice of obtaining the results in meaningful form (%) or merely in the form of a red or green pilot light on the gun itself. The green pilot light is lit if the metal complies with the planned grade, if not the red pilot is activated. In this configuration, metal identification time is generally less than 10 s.

*Jobin Yvon, the manufacturer, can be contacted at 16–18 Rue de Canal, 91163 Longjumeau Cedex, France.*

**Circle No. 113 on Reader Enquiry Card**

### 'Compromise isn't good enough'

Such is the title of a brochure about microbore HPLC from LDC/Milton Roy. The leaflet details the purpose-built system components for microbore HPLC, which include the microMetric pump and spectroMonitor D detector with a microCell fluid cell.

Microbore HPLC offers the promise of higher sensitivity and more resolution at very low flow rates, permitting solvent consumption to be as much as 90% less than with conventional HPLC. LDC/Milton Roy believe that adaption of existing HPLC instrumentation would be an unsatisfactory compromise.

*The brochure about the LDC/Milton Roy high-performance microbore system is available from Milton Roy House, High Street, Stone, Staffordshire ST15 8AR, UK. Tel.: 0785 813542.*

**Circle No. 114 on Reader Enquiry Card**

### IBM's scientific 16-bit micro

Kemtronix are launching the IBM CS-9000 scientific 16-bit microcomputer which has multi-channel chromatography software as a feature. Additional capabilities include instrument control, data acquisition, data analysis, graphics, multi-colour plotting, reports, extensive data storage and general programming. Results can be displayed on a CRT, printed as hard copy or transmitted to other computers. The micro has a resident real-time, multi-tasking operating system for scientific data handling, which may run concurrently with a XENIX operating system to provide commercial programs for word processing or laboratory management. XENIX also provides multi-user capability. A full suite of software is available including programming tools, languages, scientific subroutines, operating system extensions, graphics routines and communications protocols. The modular design of the CS-9000 allows users to tailor a system for their needs.

*Contact Kemtronix (UK) Ltd, High Street, Compton, Berkshire RG16 0NL, UK for more information. Tel.: 0653 22779.*

**Circle No. 115 on Reader Enquiry Card**

### ATE

Services for ATE (automatic test equipment) users, offered by Chippenham-based Automatic Test Engineering Ltd, are described in a brochure which is now available from the company.

ATEs are widely used in the microprocessor and electronics industries to test printed-circuit boards, subassemblies and completed products, and are generally highly sophisticated pieces of equipment requiring skilled and fast reprogramming to suit production-line changes.

Traditionally, programming has been carried out by either the ATE manufacturer or his software agent, but Automatic Test Engineering now offer an independent programming service for ATE equipment of amy manufacture either on-site or in their computer studio. According to the brochure, the advantages of independent programming include faster response, less cost and a more personal service.

In addition to program writing, the company will also design, build and commission customized ATE hardware and software to suit particularly specialized or unusual applications. All these functions are available either practically or on a consultancy basis.

*Copies from Automatic Test Engineering Ltd, Bumpers Way, Bristol Road, Chippenham, Wiltshire SN14 6LH, UK. Tel.: 0249 655342.*

**Circle No. 116 on Reader Enquiry Card**

### Pittsburgh Conference Product Review

### THE CONFERENCE

1984's Pittsburgh Conference and Exposition on Analytical Chemistry and Applied Spectroscopy was the 35th meeting to be organized by the Society for Analytical Chemists of Pittsburgh (SACP) and the Spectroscopy Society of Pittsburgh (SSP). The event began as a somewhat informal affair, and despite its international significance it is still organized and operated by a group of volunteers dedicated to providing a forum for the exchange of ideas and the exposition of state-of-the-art instrumentation in analytical chemistry and applied spectroscopy.

Papers presented during the Technical Programme reflect trends and developments in analytical chemistry and applied spectroscopy, as well as related fields: forensic chemistry, biochemistry, pharmaceuticals, industrial hygiene, etc. Scientists from around the world present new concepts and their most recent research activities.

In conjunction with the Pittsburgh Conference, several awards are made by the sponsoring societies. The 1984 Pittsburgh Analytical Chemistry Award was presented to Dr Lloyd R. Snyder, Consultant; the Pittsburgh Spectroscopy Award to Dr Jack L. Koenig, Case Western Reserve University; the Williams-Wright Award was received by Robert J. Jakobsen, Battelle, Columbus Laboratories; the Dal Nogare Award went to Dr Hamish Small; and the first Reilly Award was presented to Dr Allen J. Bard, University of Texas at Austin.

In 1940, the first Pittsburgh Conference on Applied Spectroscopy was held to discuss industrial applications of spectroscopy. The meeting was sponsored by the Spectroscopy Laboratory of the University of Pittsburgh, at that time under the direction of Mary E. Warga. The conferences stimulated interest in all forms of spectroscopy and, in 1946, the Spectroscopy Society of Pittsburgh was founded.

The Society for Analytical Chemists of Pittsburgh was established in 1942 by a group of chemists and research analysts to provide for an interchange of ideas and information about analytical problems. The first Annual Analytical Symposium organized by the group was held in 1945. As a result of the developments in analytical chemistry, the interest in these symposia grew with each succeeding year. In 1949, the Annual Analytical Symposium was held in conjunction with an Exposition of Modern Laboratory Equipment. For the first time, 'The Pittsburgh Show' was held.

When C. Manning Davis became responsible for the development of the Pittsburgh Conference on Applied Spectroscopy, he generated the idea for a joint meeting. He discussed his proposition with both groups and convinced enough people in both organizations to have a joint conference in 1950. The venture was called 'The Pittsburgh Conference on Analytical Chemistry and Applied Spectroscopy': the first was held from 15 to 17 February at the William Penn Hotel. At this first event, 57 pages were organized into 12 sessions and the exposition started reluctantly with 10 booths, three of them taken by Fisher Scientific.

The conference stayed at the William Penn Hotel until 1967 when due to an hotel strike and the need for more spacious facilities, it moved to Cleveland, Ohio. The 19th Pittsburgh Conference and Exposition, the first in Cleveland, had 39 sessions, 270 papers, and 160 exhibiting companies. Eleven years later, in 1979, 15 000 participants availed themselves 84 sessions, 730 papers, and 390 exhibiting companies.

In 1980, in order to provide additional facilities for the continued growth, the conference moved to Atlantic City, New Jersey; in 1983 it attracted 21 728 participants and 579 exhibiting companies who used 1475 booths. The technical programme consisted of 948 papers and 17 symposia; international participation included 700 conferees and 25 exhibiting companies.

### SOME OF THE PRODUCTS

### Automated viscometer

Schott America exhibited the AVS-400 automated viscometer, which uses the capillary method of viscosity determination and automatically calculates and documents data to 1/100th of a second. The model provides both mean flow time and absolute or relative viscosity.

*Full details from Schott America, Yonkers, New York 10701, USA.*

**Circle No. 117 on Reader Enquiry Card**

### Orion Scientific

At the Orion booths at Pittsburgh, the CFA products seem to be of particular interest to *JAC* readers.

The CFA-85 automated data-handling system is reasonably priced and was designed to collect, analyse and correct data from continuous-flow systems. It requires no computer experience; the keyboard allows for program changes; there is a self-contained data print-out, CRT and cassette-tape drive. Also baseline, sensitivity and carry-over corrections are possible; post-run data can be corrected as well. The computer could be used for other projects.

Orion offer a systems brochure which describes and shows industrial, laboratory and process continuous-flow analysis systems.

*Orion Scientific Instruments Corporation is based at 25 Broadway, Box 295, Pleasantville, New York 10570, USA.*

**Circle No. 118 on Reader Enquiry Card**

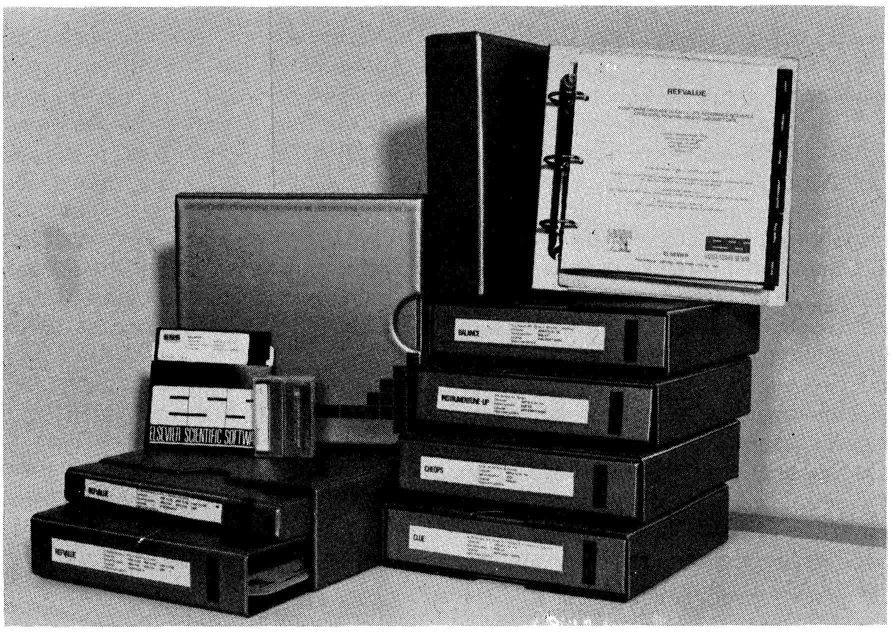

*Elsevier has entered the scientific software marked market with a series of chemical programs. Software is refereed before publication. Elsevier provide source-code listings so that the user can adapt the program to his own requirements.*

### Rapid-flow analysis

The RFA-300 system shown by Alpkem combines the advantages of flow-injection analysis and continuous-flow analysis (high speeds and low dispersion respectively). EPA- and AOAC-approved methodologies can be performed with rapid-flow analysis. The RFA-300 is a modular system with the following features:

(1) 120–240 samples/h.
(2) Micro-reagent requirements.
(3) CFA analytical cartridges with microflow path.
(4) One, two or three channel operation.
(5) Dual beam fibre-optic photometer.
(6) Self-aligning, 2 $\mu$l flowcells.
(7) 200–1200 nm solid-state photodetectors.
(8) Electronic air phasing, injection, and bubble-gating.
(9) Operates with or without optional microprocessor.

A complementary data system is available as an option from Alpkem.

*Details from Alpkem Corporation, Clackamas, Oregon 97015, USA. Tel.: 503 657 3010 or 800 547 6275.*

**Circle No. 119 on Reader Enquiry Card**

### FIAstar analyser

The new 5020 FIAstar from Tecator was designed for research and for routine chemical analyses. It allows wet-chemistry techniques to be automated. Analysis times can be from 15–60 s at a rate of 200 samples/h; typical sample volumes are in the microlitre range and reagent consumption can be maintained at less than 1–2 ml/min.

The 5020 FIAstar features replaceable manifold blocks in a compartment fitted with a thermostat, which allows for rapid changes between analytic techniques. The flow-injection system includes twin four-channel peristaltic pumps which are controlled through a microprocessor and can be operated independently, for example for stopped flow or intermittent pumping. The microprocessor is also used for calculation and digital presentation of results. Result evaluation modes include peak height, peak area, peak width (for FIA titrations) and peak-to-peak (for kinetic and stopped-flow concentrations).

Standard concentration values can be entered with function keys, which can also be used to select linear or non-linear evaluation.

*Additional information from Tecator AB, Box 70, S-263 01, Höcanäs, Sweden.*

**Circle No. 120 on Reader Enquiry Card**

### Software

Elsevier Science Publishers launched four scientific software packages at the Pittsburgh Conference. This first series deals with analytical and clinical chemistry. The programs were:

*REFVALUE*—for calculating reference intervals from hospital patients' laboratory data (authors: H. Baadenhuysen and J. C. Smit, Radboud Hospital, Nijmegen, The Netherlands). The package is primarily intended for use in clinical chemistry, but it can also be applied in other areas. It offers the possibilty of estimating reference intervals for a particular parameter, on the basis of the total bulk of unselected laboratory results. It is available for use on PDP minicomputers and the HP-85 microcomputer.

*BALANCE*—for comparing two series of measurements (authors: D. L. Massart *et al.*, University of Brussels, Belgium), for Apple II and IBM-PC micros. The purpose of the program is to determine whether two series of observations can be considered to be significantly different. The program contains several tests, parametric and non-parametric, to investigate whether, for instance, two medical treatments, two drugs, or two analytical methods yield the same result. The program is intended for non-specialists in statistics, and user-friendliness is emphasized.

*CLEOPATRA – Chemometrics Library: an Extendable Set of Programs as an Aid in Teaching, Research and Applications* (authors: G. Kateman *et al.*, University of Nijmegen, The Netherlands)—a software program for graduate and post-graduate education in chemometrics. It consists of a driver program and up to 12 modules; each of the modules explains, with the aid of very advanced graphics, a particular subfield of chemometrics (curve-fitting, filtering, time-series analysis, experimental design). Modules can also be used for practical applications. This package is now available for the HP 9845B and will soon be ready for the IBM-PC.

*INSTRUMENTUNE-UP*—an experimental optimization program (for the Apple II and IBM-PC), which helps the user to improve the performance of common scientific laboratory instruments (authors: S. N. Deming and S. L. Morgan, University of Houston and University of South Carolina, USA).

*Information about the programs from Elsevier Scientific Software, PO Box 330, 1000 AH Amsterdam, The Netherlands. Tel.: 020 5803 447.*

**Circle No. 121 on Reader Enquiry Card**

